# Changes in trophic structure of an exploited fish community at the centennial scale are linked to fisheries and climate forces

**DOI:** 10.1038/s41598-022-08391-x

**Published:** 2022-03-12

**Authors:** Leonardo Durante, Stephen Wing, Travis Ingram, Amandine Sabadel, Jeffrey Shima

**Affiliations:** 1 Department of Marine Science, University of Otago, PO Box 56, Dunedin, New Zealand; 2 Department of Zoology, University of Otago, PO Box 56, Dunedin, New Zealand; 3 School of Biological Sciences, Victoria University of Wellington, PO Box 600, Wellington, New Zealand

**Keywords:** Ecological networks, Marine biology

## Abstract

Understanding how marine food webs are affected by anthropogenic stressors is an important steppingstone toward the improved management of natural resources. Stable isotope analysis of historical and modern samples spanning a century indicated that the niche width of an exploited fish community increased after the expansion of New Zealand fisheries. Since the 2000s most species increased their reliance on food webs supported by pelagic production, compared to coastal production supported by macroalgae, and shifted to a higher trophic level. Overall changes were coincident with ocean warming, climate oscillations, prey abundance and fishing intensity, but their effects were specific to each fish assemblage analyzed. Data derived from historical samples revealed how anthropogenic stressors can drive long-term shifts in the trophic structure of an exploited fish community.

## Introduction

Complex marine food webs support global fishery catches^1,2^ and are essential for ecosystem functioning and food security^3,4^. Accelerating anthropogenic stressors, such as climate change and fisheries exploitation, have driven extensive changes in the abundance^5^ and distribution^6^ of marine organisms. While these effects have altered community composition^7^, little is known about their long-term impacts on the trophodynamics of marine food webs in natural systems.

Previous studies have demonstrated that ocean warming can alter the structure and drive the collapse of marine trophic pyramids^8–13^. Warming can increase the metabolic costs within food webs, particularly for predators and broad-spectrum omnivores, and thereby reduce fluxes of organic matter and nutrients through food webs^8,13^. Increases in trophic level and in reliance on pelagic organic matter production have been linked to higher bioenergetic costs of fish communities in New Zealand^14^, which can lead to reduced fish biomass. Changes in the rates of biomass transfer through marine food webs have also been linked to overfishing, especially of top predators^15^. However, these conclusions have relied heavily on mesocosm experiments and modeling of present and future climate scenarios, with baseline measurements and control treatments typically coming from already degraded ecosystems^16,17^. Examples from natural systems are rare but are essential if we are to understand the broader implications of natural processes and human activities for marine food webs^18^. For instance, overexploitation and ocean warming have had increasing influences on marine communities and ecosystem function over the last century^7,19,20^, with cumulative impacts observed across more than half of the global ocean^21^. In this context, long-term studies that incorporate archival samples to measure changes in trophic structure can provide important new insights into how the relatively recent history of human impacts have modified natural marine food webs^22–24^.

**Figure 1:**
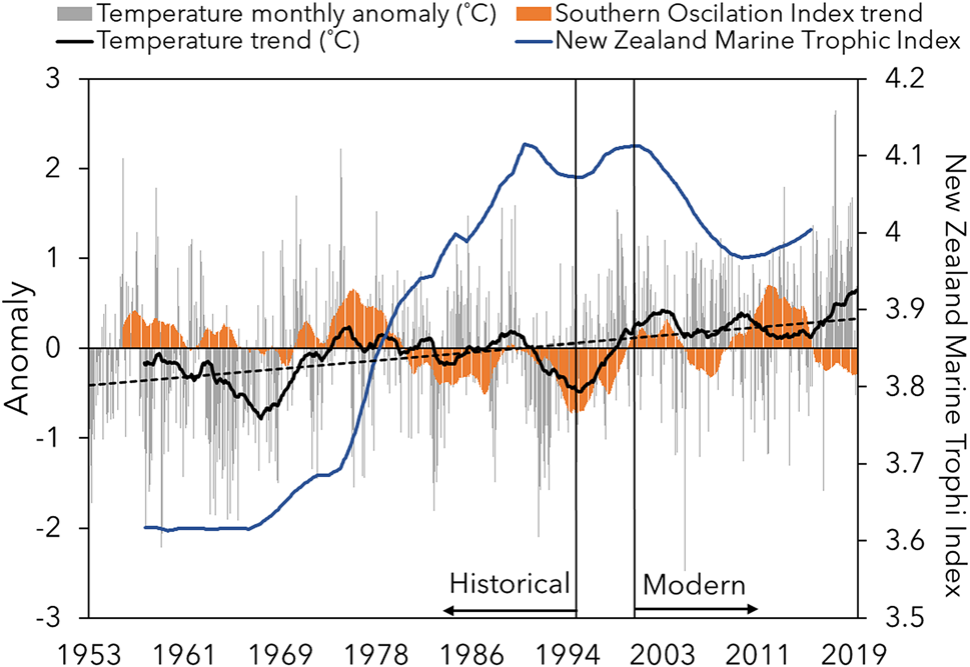
Temporal trends of environmental data and fisheries activities. Temporal trend of the Marine Trophic Index for commercial fisheries in New Zealand waters^22^, Southern Oscillation Index (SOI), monthly temperature anomaly and its temporal trend (5 year average low pass filter)^23^. SOI represents the fluctuation of atmospheric pressure in the edges of the tropical Pacific Ocean, where large negative and positive values indicate El Niño and La Niña events, respectively. Dashed line represents the linear regression of monthly temperature anomaly over the years. Straight black lines represent historical (prior to 1996), and modern (after 2000) time periods analyzed in the present study. Fish samples were not available between the years 1996 and 2000.

New Zealand's relatively recent history of European settlement, which expanded in the early 1800’s, and later fisheries industrialization provide an unparalleled opportunity to track the effects of anthropogenic impacts on its marine ecosystems^25^. Although Māori established in New Zealand at least 700 years ago^26^, the level of fisheries exploitation before widespread European settlement is estimated to be significantly lower than that after the industrialization period^27^. These systems have undergone dramatic changes in recent decades^28^, with expansion and intensification of industrialized fisheries between 1970 and 2000^22^ and increasing frequency of positive ocean temperature anomalies since 1996^23^ (Fig. 1). The system provides an ideal opportunity to estimate the effects of accelerations in these important anthropogenic stressors with changes in the trophic structure of large marine communities. For example, while worldwide marine fisheries landings have not shown significant increases between 1970 and 2018^4^, New Zealand’s landings have increased threefold during the same period^22^.

We measured naturally occurring stable isotope values from 16 exploited fish species collected from southeastern New Zealand between 1919 and 2018 (total N = 658; Table 1; Fig. S1). Stable isotopes of nitrogen (*δ*^15^N) are routinely used to estimate trophic level in ecological studies, while stable isotopes of carbon (*δ*^13^C) can be applied to detect differences in basal organic matter sources supporting species and communities^29^. We used *δ*^13^C and *δ*^15^N from muscle tissue and from specific amino acids to investigate temporal shifts in niche space occupancy, trophic level, and resource use by exploited fish species. Because the species analyzed are trophically linked^30–34^ and interact with multispecies fisheries in the region^35^, the array of species analyzed will be referred to as a community hereafter. The community was also divided into habitat-specific assemblages for further analysis.

**Table 1:** Sample sizes.

Species	Region	Historical	Modern	Total
Blue cod	All	12	58	70
*Parapercis colias*	Canterbury Bight	6	30	36
	North of Banks Peninsula-Shallow		9	9
	Marlborough Sounds	6	19	25
Elephant fish	All	15	5	20
*Callorhinchus milii*	Canterbury Bight	13	5	18
	North of Banks Peninsula-Shallow	1		1
	Marlborough Sounds	1		1
Gurnard	All	20	25	45
*Chelidonichthys kumu*	Canterbury Bight	17	24	41
	North of Banks Peninsula-Shallow	2		2
	Marlborough Sounds	1	1	2
Leatherjacket	All	23	26	49
*Meuschenia scaber*	Canterbury Bight	20	20	40
	North of Banks Peninsula-Shallow	3		3
	Marlborough Sounds		6	6
Barracouta	All	8	15	23
*Thyrsites atun*	Canterbury Bight	5	11	16
	North of Banks Peninsula-Shallow	1		1
	Marlborough Sounds	2	4	6
Common warehou	All	8	11	19
*Seriolella brama*	Canterbury Bight	6	11	17
	Marlborough Sounds	2		2
Giant stargazer	All	16	17	33
*Kathetostoma giganteum*	Canterbury Bight	7	17	24
	North of Banks Peninsula-Deep	2		2
	North of Banks Peninsula-Shallow	2		2
	Marlborough Sounds	5		5
Red cod	All	24	42	66
*Pseudophycis bachus*	Canterbury Bight	7	19	26
	North of Banks Peninsula-Deep	14	8	22
	North of Banks Peninsula-Shallow	2	14	16
	Marlborough Sounds	1	1	2
Spiny dogfish	All	15	17	32
*Squalus acanthias*	Canterbury Bight	12	17	29
	Marlborough Sounds	3		3
Tarakihi	All	21	62	83
*Nemadactylus macropterus*	Canterbury Bight	15	28	43
	North of Banks Peninsula-Shallow	6	31	37
	Marlborough Sounds		3	3
Hapuka	All	8	12	20
*Polyprion oxygeneious*	Canterbury Bight	4	7	11
	North of Banks Peninsula-Deep	1		1
	North of Banks Peninsula-Shallow	1		1
	Marlborough Sounds	2	5	7
Ling	All	20	27	47
*Genypterus blacodes*	Canterbury Bight	17	8	25
	North of Banks Peninsula-Deep	1	11	12
	Marlborough Sounds	2	8	10
Sea perch	All	32	39	71
*Helicolenus percoides*	Canterbury Bight	24	24	48
	North of Banks Peninsula-Deep	1		1
	North of Banks Peninsula-Shallow	1	11	12
	Marlborough Sounds	6	4	10
Hoki	All	11	7	18
*Macruronus novaezelandiae*	Canterbury Bight	4		4
	North of Banks Peninsula-Deep	7	7	14
Lookdown dory	All	18	12	30
*Cyttus traversi*	Canterbury Bight	6		6
	North of Banks Peninsula-Deep	12	12	24
Orange roughy	All	18	14	32
*Hoplostethus atlanticus*	Canterbury Bight	6		6
	North of Banks Peninsula-Deep	12	14	26
Total		269	389	658

Sample sizes for all species collected in different regions during historical and modern time periods.

Data analysis focused on two important themes: (1) comparing the trophic structure of modern and historical fish communities and (2) investigating the coherence of changes in environmental and anthropogenic variables with changes in the trophic structure of the fish community through time. Trophic level and resource use of historical fishes were compared with expected values from modern samples, calculated for each species using general linear models (see “Methods”; Supplementary Materials). These models accounted for differences in fish size and latitude of sampling, which are known to affect isotope values of consumers in the region^36,37^. While most species were widespread throughout the study region, species inhabiting the slope and mid shelf were primarily collected north of latitude 44° South. We evaluated long-term shifts in trophic structure of the community, based on samples collected in a large marine habitat, and their relationship with occurrence of the pelagic crab, *Munida gregaria,* Munididae (MUN; a key prey species), the Southern Oscillation Index (SOI), sea surface temperature (SST) and the Marine Trophic Index (MTI; a measure of mean trophic level of commercial fishing landings) for New Zealand waters. When aggregations of the pelagic phase of *M. gregaria* occur in the east coast of the South Island, they contribute substantially to the diet of many commercial fishes in New Zealand (including reef-associated, pelagic, and deep-water species), serving as an important link between trophic levels that span benthic and pelagic ecosystems^30,38,39^. Because of its importance, the occurrence of *M. gregaria* should be evaluated when studying the trophic ecology of important predators. On the other hand, MTI has been linked to the expansion of industrialized fisheries into new fishing grounds, increases in the amount of fishery landings and the total number of species exploited in the region^22^, and therefore serves as a general proxy for intensity of fisheries activities.

## Results

**Figure 2:**
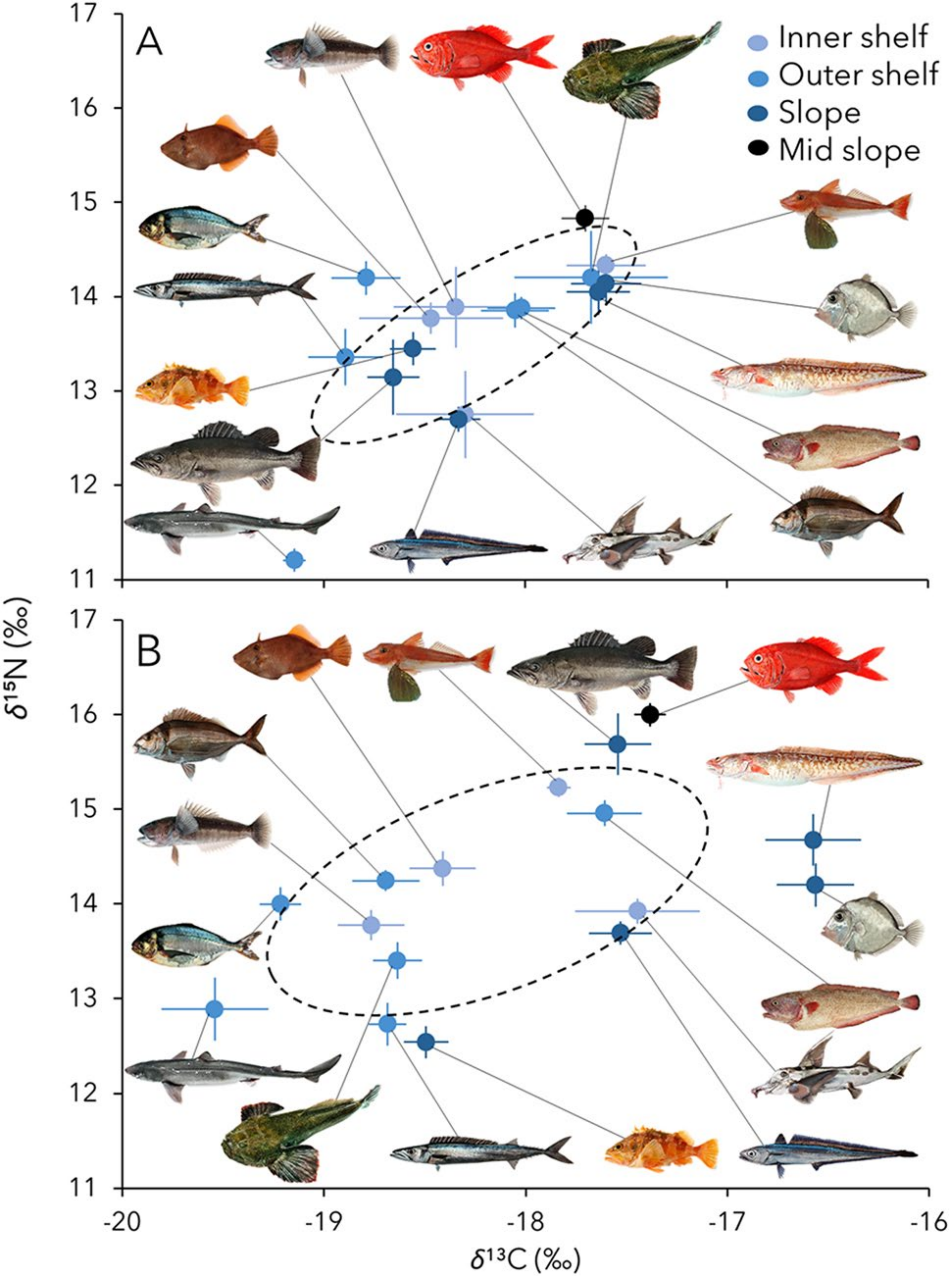
Isotopic niche spaces. Average ± standard error of the *δ*^13^C and *δ*^15^N of fish species comprising different assemblages and sampled during historical (A, before 1996) and modern (B, after 2000) periods. Dashed ellipse represents the Standard Ellipse Area of all data points calculated through Bayesian inference, representing the total niche space of the community^84^. Species are color coded by assemblages.

Modern fish communities sampled since the peak of fisheries expansion in the year 2000 displayed larger niche widths and greater interspecific trophic diversity than communities sampled before 1996 (Fig. 2). These results were primarily driven by increases in niche width and trophic diversity of outer shelf and slope assemblages (Fig. 2; Table S2). Large ranges of trophic level inferred from *δ*^15^N values were found in the deep-water species inhabiting the edge and break of the continental shelf (3.15‰—slope assemblage) (Fig. 2; Table S2).

**Figure 3:**
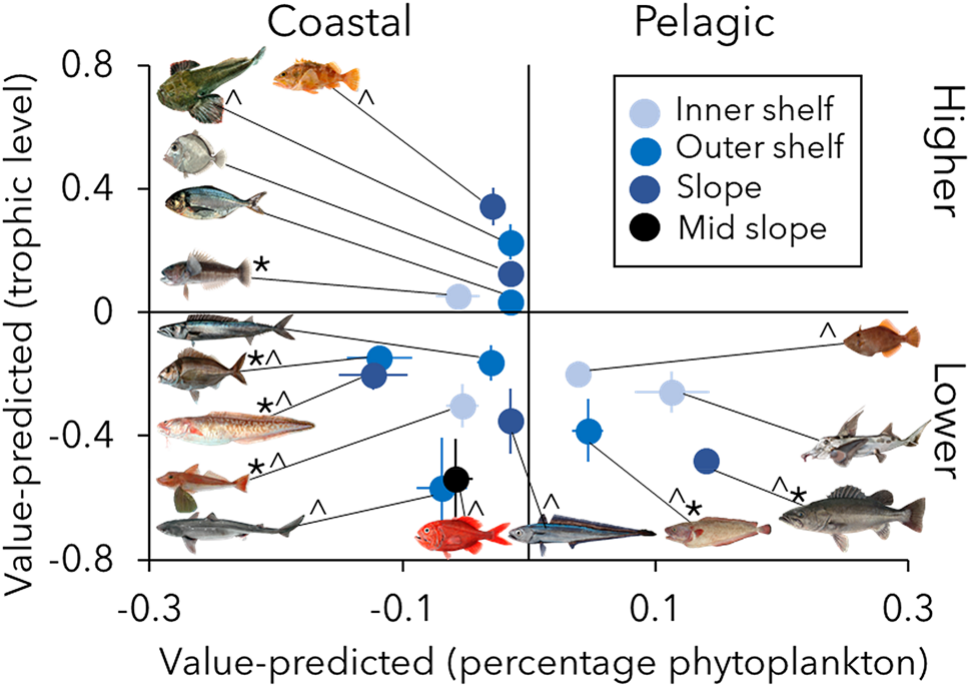
Trophic structure anomaly through time. Average ± standard error of the value-predicted percentage phytoplankton supporting the food webs and the trophic level of fishes during the historical period (before 1996) in New Zealand. Predicted values were calculated from the species-specific relationship between each trophic parameter, latitude of sampling and specimens’ total length for specimens collected after the year 2000 (modern specimens, Table S5). Panels separate species with higher and lower trophic level, as well as relying on food webs supported by more pelagic or coastal producers compared to modern predictions (i.e., historical value minus modern predicted value). Symbols represent significant differences in value-predicted percentage phytoplankton (*) and trophic level (^) between each period and modern time period (Table S3).

Trophic level estimates obtained from isotope values of muscle tissue and independently from specific amino acids provided a robust long-term record of change in the marine food web (Fig. S2). Due to cost, analysis of specific amino acids was only conducted for a subset of species and samples. Nevertheless, the consistency between methods demonstrated that isotope values from basal organic matter sources were adequate for estimating trophic level from isotopic data of muscle tissue. Trophic level and/or the proportional reliance on food webs supported by pelagic (phytoplankton) relative to benthic (macroalgae) production were different between the two time periods for many of the species analyzed. Large shifts among time periods were observed for ling (0.12% mean increase in phytoplankton reliance), tarakihi (0.12% mean increase in phytoplankton reliance), spiny dogfish (mean trophic level increase of 0.57) and orange roughy (mean trophic level increase of 0.54) (Fig. 3; Table S3). Exploited fish communities shifted to greater reliance on pelagic production over the past century, as evidenced by a smaller contribution of phytoplankton to food webs before 1996 than expected from modern data (Fig. 3). Trophic levels of most key exploited fish species were significantly lower before the full expansion of industrialized fisheries than in the modern period (Fig. 3; Table S3).

The fraction of pelagic production in food webs supporting exploited fishes had a positive relationship with SOI, with no significant variability among species (Table 2). These results reflected the relationship between contribution of phytoplankton to food webs and long-term oscillations in the Pacific weather pattern, especially for fish assemblages from the continental slope and outer shelf (Table S4). The average trophic levels occupied by the community decreased with increased pelagic prey abundance (MUN) and fisheries exploitation (MTI), while they increased with ocean temperature (SST) with significant variability among species (Table 2). Pelagic prey abundance varied together with trophic level of fishes from the continental shelf and mid slope, whereas temperature and SOI were more reliable predictors of changes in intraspecific trophic level for the assemblage from the slope habitats (Table S4). Increases in fisheries exploitation was positively correlated with intraspecific trophic level for outer shelf, slope, and mid slope assemblages. Individual species had common responses to each potential driver when grouped into assemblages (Table S4).

**Table 2:** Temperature and environmental effects.

	Percentage phytoplankton	p value	Trophic level	p value
	Value—predicted (± SE)		Value—predicted (± SE)	
**All species**	n = 606		n = 609	
Intercept	− 0.006 ± 0.009	0.562	− 0.138 ± 0.035	**0.0008**
MUN	0.0002 ± 0.0003	0.517	− 0.003 ± 0.0008	** < 0.0001**
SST	− 0.004 ± 0.012	0.721	0.184 ± 0.033	** < 0.0001**
SOI	0.073 ± 0.012	** < 0.0001**	− 0.164 ± 0.032	** < 0.0001**
MTI	0.057 ± 0.044	0.196	− 0.711 ± 0.116	** < 0.0001**
Random effect (species)	0.089		**0.019**	
Adjusted R^2^	0.12		0.24	

Results of generalized linear models (estimates ± SE) describing the effects of abundance of pelagic prey (MUN), temperature (SST), Southern Oscillation Index (SOI) and fisheries (MTI) on value—predicted percentage phytoplankton supporting food webs and trophic level for the whole fish community. Significance of species random effects are also presented in the form of p values. Value—predicted represents the differences of historical trophic parameters to expected values considering fish species, latitude, and total length (Fig. 3).

Significance values are given in bold.

## Discussion

Reductions in overlap and redundancy within food webs have been linked to mesopredator release as a result of declines in top predator abundance, associated with fishing down marine food webs^19^. The New Zealand exploited fish community had a distinct decrease in isotopic trophic redundancy (i.e. less similar isotopic niches between species and higher trophic diversity) after the full expansion of industrialized fisheries in the 2000s, characterized by high MTI (Marine Trophic Index) values (Fig. 2). Reduced specialization within the community could also indicate a less productive system with prey becoming less abundant^14,19^. Decreases of MTI after the industrial expansion period indicated reduced abundance of higher trophic level species^22^. For example, analyses of research trawl surveys from 1991 to 2018 have shown a reduction in the average trophic level of the fish community over time along the east coast of the South Island of New Zealand, but those changes were related to an increase in biomass of intermediate trophic level species^40^. Decreased redundancy in food webs and the recent increase in abundance of intermediate trophic level species are consistent with an earlier decrease in the biomass of high trophic level species and the mesopredator release from predation in the region^19,40,41^. The expansion of New Zealand’s industrialized fisheries resulted in exploitation of new offshore and deep-sea grounds^22^, where the largest decreases in trophic redundancy were observed (Table S2), indicating the spatially heterogeneous effects of fisheries on the food web dynamics of exploited species.

Larger ranges of *δ*^13^C values in the modern period were consistent with an increase in the diversity of alternate basal organic matter sources supporting food webs. The pattern can result in fundamental changes in food web structure, such as loss of high order consumers in inflexible trophic architectures due to anthropogenic stress, as discussed by Nagelkerken et al*.*^9^*.* In contrast to observations from mesocosm experiments^9^, species in the present study displayed greater trophic flexibility (Fig. 3), increasingly relying on food webs supported by pelagic production and feeding at higher trophic levels during the modern time period. While relationships between resource use and environmental variables were similar for all fishes in the present study, changes in individual species’ trophic levels reflected interspecific variation in plasticity and/or stressor exposure (Table 2). Therefore, sources of organic matter supporting fisheries food webs were more affected by oscillatory environmental forces, while species-specific trophic level was closely tied to the trophic ecology of each species, affected by both environmental and anthropogenic stressors. The observed variability might be specific to each fish habitat or depth, since species effects were non-significant when assemblages were analyzed separately (Table S4).

Long-term warming and marine heatwaves have been correlated with increased pelagic production and local reduction of kelp beds in the study region^42,43^, likely resulting in the observed shift in basal organic matter sources supporting marine food webs. The increase in trophic level may have been driven by competition for limited resources leading species to feed upon other secondary consumers^9^. For example, Pinkerton et al.^44^ demonstrated that trophic levels of fishes are likely to increase with decreases in their abundance in New Zealand. Observed intraspecific increases in trophic level between time periods in the present study were coincident with decreases in abundance for many of the key species analyzed^35^ (Fig. 3).

The fish community fed at lower trophic levels during years with more intense fishing activities characterized by high MTI values (Table 2). Exploitation has been shown to drive high trophic level species to shift prey fields, lowering their trophic level and altering the flux of energy through food webs in overexploited regions^45–49^. An increase in fish trophic level has been reported due to a decrease in consumer biomass in New Zealand^44^, but this was not observed in the present study when only MTI was considered in the analysis. The study by Pinkerton et al*.*^44^ was also conducted within a gulf, displaying lower number of fish habitats and less external influences, such as fish movement, compared to the present study region. Fishing activities impacted the trophic level occupied by fish species primarily in the outer shelf, slope and mid slope assemblages (Table S4), which were the habitats most affected by exploitation during the time frame of the present analysis^22^. Although overexploitation was only identified for a few stocks over the period and in the region of the present study^50^, exploitation rates have decreased since the introduction of the Quota Management System and other restrictions to international fleets^22,51^.

Although positive and negative SOI values indicated warmer La Niña and colder El Niño conditions in New Zealand, respectively, SOI had the reverse effect of SST on intraspecific trophic levels (Table 2; Table S4). Furthermore, fishes collected during years of positive SOI had a higher reliance on food webs supported by pelagic production. El Niño Southern Oscillation (ENSO) can impact the diversity and biomass of pelagic prey available to fish species^52^, therefore modifying their trophic ecology. For example, the diet of hake (*Merluccius gayi peruanus*) was shown to be more diverse during warmer El Niño events in the Humboldt Current ecosystem, but feeding activity and/or food supply was more heterogeneous^53^. The effects of ENSO on nutrient availability and pelagic primary production are still poorly understood in New Zealand^54^. However, increased northward transport of nutrients and average fluorescence in the water column were observed in colder years throughout the study region of the present study^37^. Warmer years with positive SOI values could have altered the biomass and diversity of the lower levels of the pelagic food web in the east coast of the South Island, which might have resulted in species feeding at lower trophic levels. Furthermore, the observed pattern also indicates that along with the overall warming trend, oscillatory changes in temperature and rainfall, likely linked to marine heatwaves, can also influence trophic structure of fisheries food webs.

When abundance of the pelagic primary consumer *M. gregaria* (MUN) is high, pelagic prey can represent a large proportion of the diet of the exploited fish species in southeastern New Zealand^30,55^, decreasing a consumer’s trophic level (Table 2). Reduction of MUN abundance over time was therefore linked to the overall increased trophic level of fish species. The pattern was observed for most fish assemblages (Table S4) and highlights the importance of *M. gregaria*, for maintenance of the food web supporting commercial fish species in New Zealand. Reduction in MUN abundance over time could also have contributed to the decreased trophic redundancy observed in the present study, since in the absence of large aggregations of MUN fish species would likely consume different prey items at contrasting trophic levels and supported by alternate basal organic matter sources. The frequency, size and duration of MUN aggregations in the pelagic system is thought to be positively correlated with the number of adults occupying the benthos, with high numbers hampering cohort settlement from the pelagic system, but can also be related to environmental conditions^55,56^. Therefore, low number of MUN aggregations in the most recent years could be related to low overall biomass of MUN in the regions and poor environmental conditions, such as higher temperature and lower pelagic production.

Due to sample availability, especially from the historical period, the comparison between modern and historical ecological states is sample rich but coarse in temporal resolution, while continuous analysis of environmental and fisheries data presents high year-to-year resolution but low replication. The dissimilarity can result in contradictory results depending on when samples were collected. For example, while exploitation was related to a decrease in trophic level for the community of caught species, many individual fish species had increases in trophic level coincident with increases in fisheries activities throughout the whole period. The observed pattern was influenced by frequency of samples collected during the peak of fisheries expansion, with a lack of samples collected between the year 2000 and 2012. However, both results are supported by the available data.

Ocean warming can cause shifts in productivity and biomass in communities with relatively inflexible trophic architecture, which can strongly reduce stability of marine food webs^9,13^. Furthermore, from a modeling approach, overfishing has been shown to change species composition of fish communities and alter marine food webs^15,57^. Shifts in trophic architecture such as the ones observed in the present study were previously linked to increased energy cost of species occupying trophic levels and relying more on pelagic compared to coastal production^14,58^. When coupled with decreased pelagic production and increased metabolic rates of fishes due to ocean warming, these results likely indicate a reduction in marine fisheries production in the future. Globally, most coastal areas have experienced increases in fishery activities compared to historical baselines^59^. The present study highlights the importance, and provides a framework, for considering these historical patterns to understand current ecological states in exploited marine ecosystems.

The present study provides evidence for long-term changes in the trophic structure of an exploited fish community. The present study was based on specimens collected over a 100 year period in a large marine habitat during times with contrasting temperature regimes and fishing intensity. Continuous changes in trophic structure were related to environmental, biological, and anthropogenic stressors, and were manifested differently among coastal, shelf and continental slope fish assemblages. While ocean warming alone may not affect the trophic structure of consumers^9^, shifts in trophic architecture are a possible outcome in natural systems affected by changing exploitation, prey availability and climate^60^, with important consequences for trophodynamics in fisheries ecosystems.

## Methods

### Sample collection

Sixteen species of fish were collected along the east coast of the South Island between 2017 and 2018 (Table 1, Table S1). Coastal species were collected during scientific cruises onboard the RV *Polaris II* or provided by recreational anglers, while fishes that inhabit offshore waters were mainly provided by commercial fishers or from the Canterbury Bight and Pegasus Bay trawl survey^61^. Deeper water species, such as lookdown dory (*Cyttus traversi*, Cyttidae), orange roughy (*Hoplostethus atlanticus*, Trachichthyidae) and hoki (*Macruronus novaezelandiae*, Merlucciidae) were acquired from the Chatham Rise trawl surveys^62^. Specimens collected onboard research vessels were measured for total length and head length and were frozen and taken to the laboratory to sample muscle tissue. Since samples provided by fishers often consisted of fish heads only, a linear regression between total length and head length was calculated, to estimate total length of those specimens and include them in the main analysis (Table S6).

To access the stable isotope values and estimate trophic parameters of fish species before the present date, specimens were retrieved from the Otago Museum and the Museum of New Zealand Te Papa Tongarewa collections, spanning 1919–2012, most comprising the “historical” sample group (period between 1919 and 1996). Muscle tissue (1 cm^3^) was sampled from the dorsal musculature of all specimens for isotope analysis. Tissue samples from museum collections were then treated with deionized water for 1 week to remove excess preservatives (mainly ethanol and isopropanol) before analysis^63^. Temporal changes in the environment and fisheries activities have marked the separation between historical and modern periods, allowing the present study to investigate its effects on the trophic structure of fish communities. While the historical period was marked by the development and extension of New Zealand fisheries and relatively colder temperatures, New Zealand fisheries have reached their peak and sea surface temperature anomalies have presented a steady increase during the modern period^22,23^ (Fig. 1).

Estimates of trophic level based on isotopic analysis rely on knowledge of isotope values of the primary producers supporting food webs. Coastal (macroalgae), and pelagic (phytoplankton) primary producers were assumed to be the main contributors of organic matter to the fish communities within the study region and used as isotope baselines in the present study. *δ*^13^C and *δ*^15^N values of macroalgae were obtained from Otago (− 15.93 and 8.1‰), Kaikoura (− 13.46 and 8.22‰) and the Marlborough Sounds (− 14.86 and 6.84‰) (^48^ and unpublished data). *δ*^13^C and *δ*^15^N values of phytoplankton were inferred from samples of suspended particulate organic matter (SPOM) collected during oceanographic cruises inside the Canterbury Bight (− 24.00 and 5.45‰), along the Kaikoura coast (− 21.1 and 7.39‰) and Marlborough Sounds (− 22.75 and 6.29‰)^37,48^. Both macroalgae and SPOM samples were collected concomitantly with the collection of specimens in the modern period.

### Sample preparation and bulk analysis

Muscle tissues were oven-dried at 60 °C for 72 h and ground to a fine and homogenous powder with mortar and pestle. All equipment was cleaned with low-linting Kimwipes^®^ and ethanol and air-dried between samples to avoid cross-contamination. Between 0.8 and 1.2 mg of each sample was packed in 3 × 5 mm tin capsules and analyzed by combustion in an elemental analyzer (Carlo Erba NC2500) coupled with a Europa Scientific 20–20 ANCA Mass Spectrometer operating in continuous flow. Delta values were normalized and reported against the international standards Vienna Pee Dee Belemnite (VPDB) and atmospheric N_2_ (AIR) for *δ*^13^C and *δ*^15^N, respectively. The ratio between the molar amount of carbon and nitrogen in each sample (C:N) was also reported. Normalization was made by 3-point calibration with two glutamic acid international reference materials and a laboratory EDTA standard (Elemental Microanalysis) for carbon (USGS-40 =  − 26.2 ‰, USGS-41 = 37.8 ‰, EDTA = − 38.93 ‰) and nitrogen (USGS-40 =  − 4.52 ‰, USGS-41 = 47.57 ‰, EDTA = − 0.73 ‰). Analytical precision was checked by comparing results from analyzed quality control standards EDTA-OAS and IAEA MA-A-1 (Copepod) against accepted values. All measured values for the quality control standards were in the range of accepted values. Additionally, one sample of fish muscle tissue was analyzed in every run so results could be corrected for in-between run variability.

Because lipids are depleted in *δ*^13^C compared to protein and carbohydrates, changes in lipid content in the samples can have a large effect in *δ*^13^C values that are not linked to trophic parameters. In the present study, the following normalization equation was applied to all samples collected during the modern period to account for variation in lipid concentrations, after Post et al.^64^:

1 $$ \delta^{{{13}}} {\text{C}}_{{{\text{Normalized}}}} = \delta^{{{13}}} {\text{C}}_{{{\text{Untreated}}}} {-}{ 3}.{32 } + \, 0.{99 }*{\text{ C}}:{\text{N}} $$

Here *δ*^13^C_Untreated_ represents raw *δ*^13^C measurements and C:N the carbon to nitrogen ratio of the muscle tissue. The normalization applied is suitable for aquatic organisms and was generated with the same range of C:N and *δ*^13^C values as found in the present study^65^. To account for the effects of fixatives and preservatives on the isotope values of muscle tissue, corrections were applied to all samples from museum collections, for both *δ*^15^N (Eq. 2) and *δ*^13^C (Eq. 3) after Durante et al.^63^ These corrections are independent of time under preservation and normalize *δ*^13^C values due to different lipid content in muscle tissue^63^. Where *δ*^15^N and *δ*^13^C_lipid free_ represent corrected values using *δ*^15^N, *δ*^13^C, proportion of N and the C:N ratio from preserved specimens.

2 $$ \delta^{{{15}}} {\text{N }} = { 11}.{25} + 0.{71} \times \delta^{{{15}}} {\text{N}}_{{{\text{preserved}}}} + 0.{27} \times \delta^{{{13}}} {\text{C}}_{{{\text{preserved}}}} {-}0.{\text{21 proportion of N}}_{{{\text{preserved}}}} $$

3 $$ \delta^{{{13}}} {\text{C}}_{{\text{lipid free}}} { = } - {8}.{42} + 0.0{7} \times \delta^{{{15}}} {\text{N}}_{{{\text{preserved}}}} + 0.{76} \times \delta^{{{13}}} {\text{C}}_{{{\text{preserved}}}} + 0.{97} \times {\text{C}}:{\text{N}}_{{{\text{preserved}}}} $$

*δ*^13^C values of primary producers have also been globally influenced by anthropogenic activities since the industrialization period in the 1950’s^66,67^, and to compare with present values those changes need to be taken into account. These processes are known to influence fishes from different trophic levels and have been reported for the Otago region^68^. The Suess effect (decrease of atmospheric *δ*^13^C of CO_2_) was corrected for^69^, which predicts a decrease in *δ*^13^C_Bulk_ of on average 0.011‰ per year (− 0.014 ± 0.001‰ to − 0.006 ± 0.001‰) in the ventilated South Pacific Ocean.

### Amino acid stable isotope analyses

To test the accuracy of trophic level estimates when primary producers could not be sampled (historical period), compound-specific stable isotope analysis of amino acids (CSIA-AA) were used^70,71^. Nitrogen isotope in different amino acids (*δ*^15^N_AA_) fractionate in contrasting ways between trophic levels and can be classified as source (no-to-little fractionation of *δ*^15^N) and trophic (large fractionation of *δ*^15^N). Consequentially, use of *δ*^15^N_AA_ allows researchers to access information regarding a specimen's trophic level from a single muscle tissue sample^72^. The technique is especially useful when isotope values of primary producers for the estimation of trophic level are not known or cannot be sampled, allowing one to investigate changes in trophic parameters at large spatial and temporal scales^73–75^. For example, the relationship between *δ*^15^N of Glutamic acid (*δ*^15^N_Glx_, trophic AA) and *δ*^15^N of Phenylalanine (*δ*^15^N_Phe_, source AA) have been used to accurately estimate trophic level of aquatic animals^71^.

Amino acids were extracted by hydrolyzing 2.5 mg of sample with 2 ml 6 M HCl at 110 °C for 24 h in a N_2_ atmosphere. An internal standard, norleucine (50 μl of 1 mg/ml solution), was added to monitor the wet chemistry and AA stable isotope values. Solutes were then dried under a gentle flow of N_2_ at 60 °C and subsequently converted into *N*-acetylisopropyl (NAIP) ester derivatives following the protocol described in^76^, modified from^77^. See Sabadel et al*.* for full methods details. *δ*^15^N_AA_ was measured by gas chromatography/combustion/isotope ratio mass spectrometer (GC-IRMS), using a Thermo Trace gas chromatograph, the GC combustion III interface, and a Deltaplus XP isotope ratio mass spectrometer (Thermo Fisher Scientific). 200 nl aliquots of derivatized AA were injected in an inlet at 270 °C in spitless mode, carried by helium at 1.4 ml min^−1^ and separated on a VF-35 ms column (30 m long, 0.32 mm ID and a 1.0 μm film thickness). Samples were analyzed in duplicates or triplicates along with amino acid standards of known isotopic composition (measured by EA-IRMS) and bracketing measurement of every two samples. Each run contained no more than 10 samples. Similar to bulk, *δ* values from AA were reported following the conventional method of expressing δ at natural abundance, in per mil (‰), relative to an international standard of atmospheric N_2_ for *δ*^15^N_AA_^78^.

Glutamine + glutamic acid (Glx) and phenylalanine (Phe) were measured from 50 samples from the historical period and comprising eight species. Note that during the hydrolysis step glutamine is converted to glutamic acid. Precision (1SD) of *δ*^15^N_AA_ ranged from 0 to 1.1‰ with a mean of 0.4‰.

### Trophic parameter calculations

Because the variability of isotopic baselines can affect the bulk isotope values of fish muscle tissue, we used region-specific baselines from the two main primary producers, macroalgae and SPOM (corresponding mainly to phytoplankton^37^), to estimate trophic level and percentage phytoplankton supporting the food webs for each fish. For the analysis, a two-step iterative procedure based on bulk isotope values was used to generate a mass balance model, following Phillips and Gregg^79^. First an approximation of the contribution of each organic matter source to a fish’s *δ*^13^C value was calculated from plotted *δ*^15^N vs. *δ*^13^C values. The results were then used to estimate the corresponding *δ*^15^N value of the mixture of organic matter sources supporting each specimen (*δ*^15^N_resource_)^80^, assuming the same contribution from the *δ*^15^N pool. Trophic level was then calculated for each fish using its own *δ*^15^N value (*δ*^15^N_Consumer_):

4 $$ {\text{TL}} = {1} + \left( {\delta^{{{15}}} {\text{N}}_{{{\text{Consumer}}}} - \delta^{{{15}}} {\text{N}}_{{{\text{resource}}}} } \right)/{\text{TDF}} $$

Because of the differences in diet, i.e. invertivore and piscivores, trophic discrimination factor (TDF) was assumed to be 3.4‰ (SD 1) for fishes mainly feeding on invertebrates and 2.3‰ (SE 0.28) for species with reported diet being composed by more than 50% fish^81,82^ (Table S1). The result of the equation was then iterated back into the mass balance model until a stable solution was obtained for both the mixture of organic matter sources and TL for each specimen. A trophic discrimination factor for aquatic environments of 0.5‰ (SE 0.17) was used for *δ*^13^C^81^. Average isotopic values of primary producers have had no significant change in the past two decades^37^, therefore they were assumed to have been the same among periods analyzed.

To test the accuracy of trophic level estimates from bulk isotopes we compared those estimates with trophic level calculated from CSIA-AA. Trophic levels based on amino acid isotope results were calculated based on differences between *δ*^15^N_Glx_ and *δ*^15^N_Phe_, following Chikaraishi et al*.*:

5 $$ {\text{TL}}_{{{\text{Glx}} - {\text{phe}}}} = {1} + \left( {\delta^{{{15}}} {\text{N}}_{{{\text{Glx}}}} - \delta^{{{15}}} {\text{N}}_{{{\text{Phe}}}} - { 3}.{4}} \right)/{\text{TDF}}_{{{\text{Glx}} - {\text{Phe}}}} $$

With 3.4‰ the difference between *δ*^15^N_Glx_ and *δ*^15^N_Phe_ in aquatic cyanobacteria and algae^71^. Similar to trophic level estimates from the mixing model, the trophic discrimination factor representing the difference in fractionation per trophic level of *δ*^15^N_Glx_ and *δ*^15^N_Phe_ (TDF_Glx-Phe_), was chosen according to the fish species’ diet^83^. In their feeding experiment study, McMahon and co-authors found that fishes fed on high protein diets, where the AA composition were similar to the fishes’ muscle tissue, presented a smaller TDF_Glx-Phe_ than fishes feeding on an omnivorous diet^83^. Therefore, in the present study a TDF_Glx-Phe_ value of 7.6‰ was used for species with fish contribution to its diet lower than 50%^71^, while a value of 5.9‰ was chosen for piscivore species^83^ (Table S1).

### Data analysis

Changes in niche width in the whole fish community and within each assemblage (see Table S1) were investigated between periods using ellipse-based versions of Layman’s metrics estimated from Bayesian inference, using the SIBER package in R^84,85^. The use of ellipse-based metrics is suitable for comparisons between communities with different samples sizes, with improved estimates and reduced uncertainty compared to original metrics. For example, total area of convex hull (TA) of the *δ*^13^C and *δ*^15^N biplot, or isotopic space, can be easily biased by large isotope values from few individuals, while the calculation of the Standard Ellipse Areas corrected for small sample sizes (SEA_c_) provides Bayesian distributions that reflect the uncertainty of estimates, resulting in more robust and less biased comparisons^84^. Similarly, the mean Euclidian distance from each species to the *δ*^15^N and *δ*^13^C centroid of the whole food web (CD) is a metric of average trophic diversity that is also affected by trophic packing^85^. Moreover, the mean nearest neighbor distance (MNND) and the standard deviation (SDNND) of the same plot can be used as proxies for trophic packing and its evenness, respectively.

Body size and latitude of sampling can influence the isotope values in fish muscle tissue and hamper investigations of isotopic niche space. For practicality, museums tend to keep only small to medium size specimens in their wet collections, creating a bias in historical samples towards small individuals. To account for those effects, before Layman’s metrics analysis, large specimens from the modern period and small from museum collections were excluded until both groups achieved equal distributions of total length (Wilcoxon test and ANOVA, p > 0.05). Likewise, isotope values of each species were only compared when samples were collected from the same latitude range in both periods, historical and modern (N = 466, Table S7).

To investigate how the trophic level and percentage phytoplankton supporting the fish community have varied were different between the two time periods, the anomalies of those parameters were calculated, by comparison to modern samples. We fitted general linear models for each species collected in the modern group, with percentage phytoplankton and trophic level as response variable, and latitude and specimen’s total length as explanatory variables. Coefficients were calculated with a least square approach, which is sensitive to outliers and performs well with noisy, collinear and incomplete variables, being suitable for analysis of complex models in chemistry and biology^86^. All combinations of variables were tested and linear models with the lowest Akaike Information Criteria corrected for small sample sizes (AIC_c_) were selected. When latitude and/or size contributed significantly to the estimate of trophic level and percentage phytoplankton, the resulting equation was used to estimate the expected percentage phytoplankton and trophic level of individuals. When latitude and total length did not contribute to the model, average values of modern species were used as predicted percentage phytoplankton and trophic level values. In both cases, groups were only analyzed when samples were collected from similar ranges of latitude and/or had similar range of total length in both periods, historical and modern (N = 622, Table S8).

The difference between real (estimated through bulk isotopes) and expected (modeled through multiple linear regression) resulted in the values-predicted percentage phytoplankton and trophic level. To investigate changes in percentage phytoplankton and trophic level of the exploited community, values-predicted were plotted for communities sampled before the full expansion of industrialized fisheries in New Zealand (before 1996, or historical). Differences in value-predicted percentage phytoplankton and trophic level between historical and modern samples were analyzed for each separate species using a PERMANOVA. Unrestricted permutations (10,000) of the dissimilarity matrices calculated with Euclidian distance were used for each variable separately. PERMANOVA is a type of analysis of variance that uses permutation to compute statistical tests instead of statistical tables, and does not assume linearity or normality^87^.

The effects of sea surface temperature (SST), Southern Oscillation Index (SOI), fisheries activities (MTI) and abundance of pelagic prey (MUN) were investigated by fitting general linear models to value-predicted percentage phytoplankton and trophic level. Yearly anomaly values of the independent variables were used in the models, considering an average between 1953 and 2018. SOI was calculated with the difference in air pressure between Tahiti and Darwin; while low SOI values indicated El Niño events, La Niña was identified by high SOI values. The east coast of the South Island of New Zealand can experience droughts during both anomalies, while La Niña is usually associated with higher temperatures^88^. SST was recorded at the Portobello Marine Laboratory of the University of Otago (PML), and although it represents a localized proxy of ocean warming, it has been reported to follow large scale processes, such as the overall increase in temperatures in boundary currents associated with changes in wind regimes^23^. MUN was inferred from the sum of the sightings of *M. gregaria* from PML in each year^39,55^, and MTI calculated for New Zealand waters was used as a proxy of the state of fisheries expansion in a given year, after Durante et al.^22^. Results from Durante et al.^22^ comprise the most accurate MTI values available for the New Zealand region, with data amalgamated from Fisheries New Zealand and the Food and Agriculture Organization of the United Nations and annual catches identified to species level. Because MTI was only calculated until 2015, the value from 2015 was used in the years 2017 and 2018. As shown by Durante et al*.*, MTI values for New Zealand have not varied greatly in the most recent years, which justifies the approximation when comparing to long-term shifts in MTI. Yearly values of SOI, SST, MUN and MTI did not show strong correlation (Pearson’s coefficient correlation from − 0.32 to 0.42) and were therefore fit for multiple linear model analysis. Species were analyzed as a whole community and as four different assemblages (inner shelf, outer shelf, slope and mid slope) according to their habitat use and composition after Francis et al*.*^89^ and Beentjes et al*.*^90^ (Table S1). All statistical analysis were undertaken with R 3.6.3^91^, JMP 14.0.0^92^ and PRIMER 6.1.12^93^.

### Ethical approval

Collection and handling of animals were approved by the University of Otago ethics committee under the protocol AUP-18-182 and were conducted in accordance with relevant guidelines and regulations. Methods were reported in accordance with ARRIVE guidelines.

## Supplementary Information

Supplementary Information.

## Data Availability

All data is available in the supplementary spreadsheets “Durante_etal_data1.xlxs”, “Durante_etal_data2.xlxs” and “Durante_etal_data3.xlxs”.
