# Supplementary material for: Changes in trophic structure of an exploited fish community at the centennial scale are linked to fisheries and climate forces
Source: Sci Rep. 2022 Mar 12;12:4309. doi: 10.1038/s41598-022-08391-x (PMC8918348; doi:10.1038/s41598-022-08391-x)
Supplement: Supplementary file 1 — Supplementary Information. [file 41598_2022_8391_MOESM1_ESM.docx]

Supplementary Materials

Changes in trophic structure of an exploited fish community at the centennial scale are linked to fisheries and climate forces.

**Authors:** Leonardo Durante^1^*, Stephen Wing¹, Travis Ingram^2^, Amandine Sabadel^1,2^ and Jeffrey Shima^3^

**Affiliations:**

^1^Department of Marine Science – University of Otago, Dunedin, New Zealand PO Box 56.

^2^Department of Zoology – University of Otago, Dunedin, New Zealand PO Box 56.

^3^School of Biological Sciences – Victoria University of Wellington, Wellington, New Zealand PO Box 600

*Correspondence to: leo_durante7@hotmail.com; +61 049 295 0859


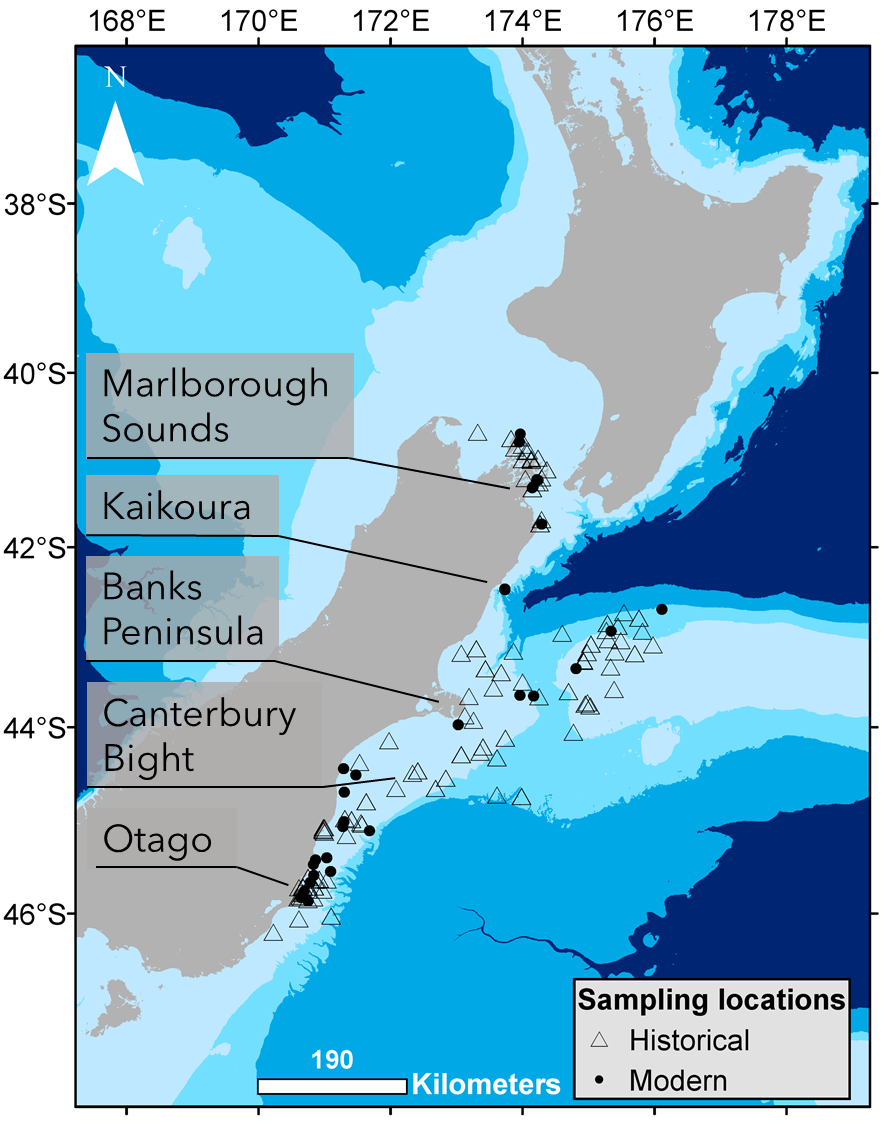


**Fig. S1. Sample locations.** Map of the New Zealand marine environment with the location of historical (1919-1996) and modern (2000-2018) fish samples analyzed in the present study. Regions of interest are shown. Grey represents New Zealand landmass and blue shades (from light to dark) identify depth bands of 0 to 500, 500 to 1000, 1000 to 2000 and deeper than 2000 meters. Map was generated with ArcGIS Desktop 10.7.1 (https://desktop.arcgis.com/en/arcmap/10.7/get-started/setup/arcgis-desktop-quick-start-guide.htm).


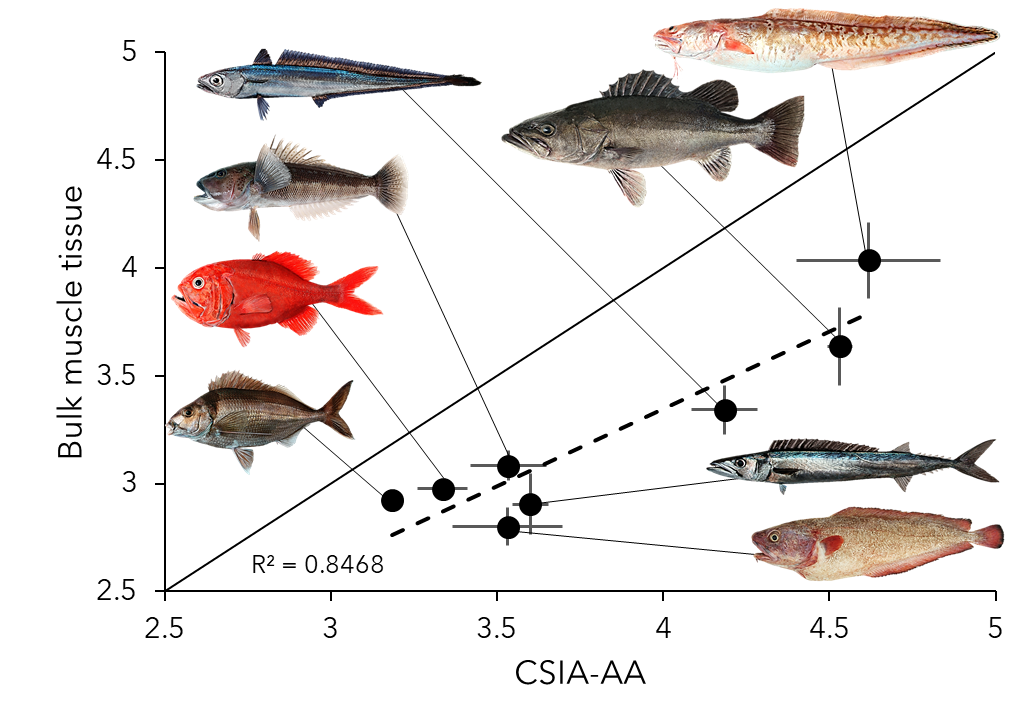


A

B

C

D

E

F

G

H

**Fig. S2. Trophic level estimates for historical samples.** Comparison between trophic level estimates (average ± SE) from bulk muscle tissue and compound-specific isotopic analysis of amino acids (CSIA-AA) for eight species of fishes sampled during the historical period (before 1996). Dashed line represents a linear regression between species estimates, while solid line is the 1:1 ratio between axes. Sample sizes: Blue cod (A, 5), barracouta (B, 6), red cod (C, 7), tarakihi (D, 8), hapuka (E, 6), ling (F, 7), hoki (G, 6) and orange roughy (H, 5).

**Table S1.**

Total length, habitat, depth distribution, assemblage group and common prey items of the fish species analyzed in the present study. Habitat data was retrieved from Fishbase.com, Beentjes *et al*. and Francis *et al*. Percentage of occurrence of food items were retrieved from Stevens *et al.*

| Species | Total length (mm) | Habitat | Common prey items | Ref. |
| --- | --- | --- | --- | --- |
| Leatherjacket *Meuschenia scaber* | 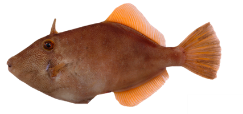310 | Inner shelf | Crustaceans | ^1^ |
|  |  | Demersal | Molluscs | ^2^ |
|  |  | 0 - 100 m | Sponges |  |
|  |  |  | Tunicates |  |
|  |  |  | Echinoderms |  |
|  |  |  | Algae |  |
| Blue cod *Parapercis colias* | 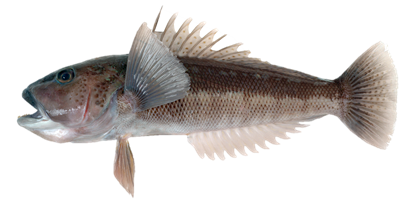600 | Inner shelf | Fish (47%) | ^3^ |
|  |  | Demersal | Crustaceans (23%) | ^2^ |
|  |  | 0 - 150 m | Tunicates (17%) | ^4^ |
|  |  |  | Cephalopods | ^5^  Personal observation |
|  |  |  | Gastropods |  |
|  |  |  | Bivalves |  |
|  |  |  | Worms |  |
| Gurnard  *Chelidonichthys kumu* | 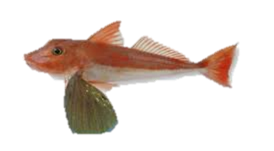550 | Inner shelf | Crustaceans (50%) | ^6^ |
|  |  | Demersal | Fish (43%) | ^5^ |
|  |  | 0 - 200 m | Worms | ^7^ |
|  |  |  |  |  |
| Elephant fish *Callorhinchus milii* | 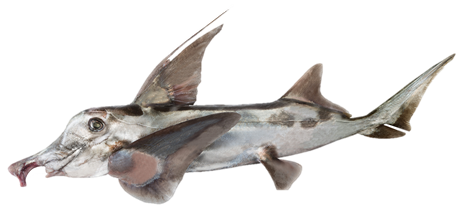970 | Inner shelf | Bivalves | ^5^ |
|  |  | Demersal | Jellyfish/hydroids | ^1^ |
|  |  | 0 - 200 m |  | ^8^ |
|  |  |  |  | ^3^ |
| Common warehou *Seriolella brama* | 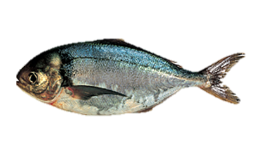750 | Outer shelf | Salps (97%) | ^9^ |
|  |  | Benthopelagic | Crustaceans | ^5^ |
|  |  | 0 - 400 m | Jellyfish/hydroids | ^7^ |
|  |  |  | Cephalopods |  |
| Barracouta *Thyrsites atun* | 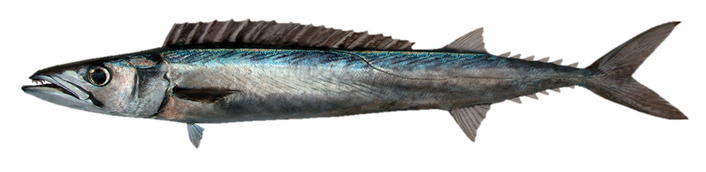2000 | Outer shelf | Crustaceans (77%) | ^5^ |
|  |  | Benthopelagic | Fish (18%) | ^3^ |
|  |  | 0 - 400 m | Squid (9%) | ^10^ |
|  |  |  |  | ^11^ |
| Tarakihi  *Nemadactylus macropterus* | 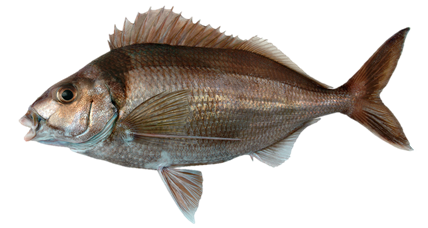700 | Outer shelf | Chitons | ^12^ |
|  |  | Demersal | Gastropods | ^3^ |
|  |  | 0 - 486 m | Bivalves | ^2^ |
|  |  |  | Crustaceans | ^13^ |
|  |  |  | Echinoderms | ^14^ |
|  |  |  | Polychaetas |  |
|  |  |  | Cephalochordates |  |
| Spiny dogfish *Squalus acanthias* | 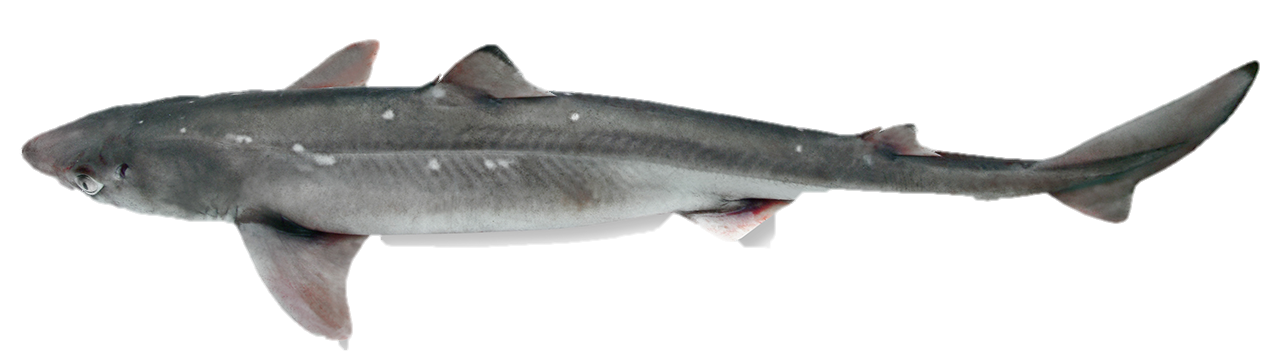1360 | Outer shelf | Fish | ^5^ |
|  |  | Benthopelagic | Squid/octopus | ^15^ |
|  |  | 0 - 500 m | Planktonic invertebrates | ^16^ |
|  |  |  | Crustaceans | ^17^ |
|  |  |  | Jellyfish/hydroids | ^18^ |
|  |  |  | Worms | ^19^ |
|  |  |  | Bivalves |  |

Continue in the next page

| Species | Total length (mm) | Habitat | Common prey items | Ref. |
| --- | --- | --- | --- | --- |
| Giant stargazer *Kathetostoma giganteum* | 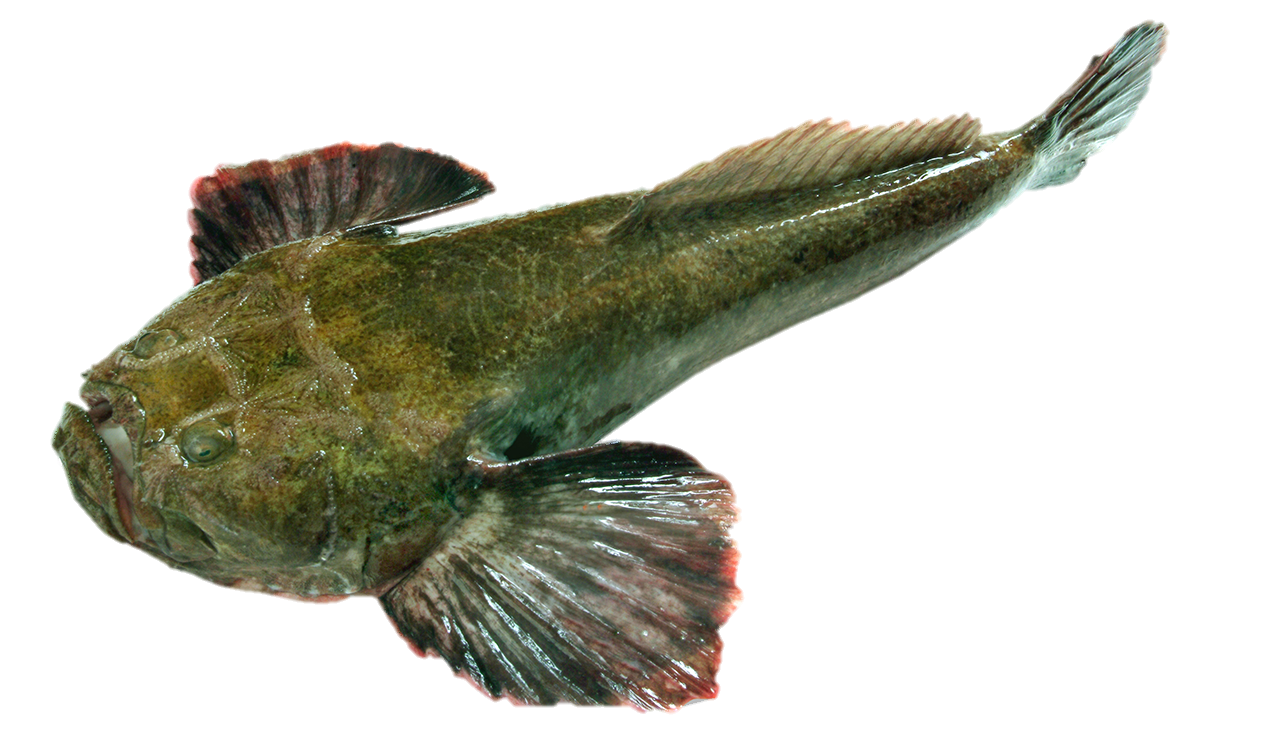780 | Outer shelf | Fish (58%) | ^20^ |
|  |  | Demersal | Cephalopods (38%) | ^21^ |
|  |  | 0 – 600 | Crustaceans (12%) | ^7^ |
| Red cod *Pseudophycis bachus* | 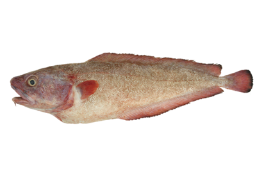900 | Outer shelf | Crustaceans (79%) | ^20^ |
|  |  | Demersal | Fish (25%) | ^22^ |
|  |  | 0 - 700 m | Molluscs | ^5^ |
|  |  |  | Worms | ^3^ |
|  |  |  | Echinoderms | ^23^ |
| Sea perch *Helicolenus percoides* | 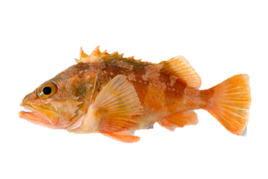470 | Slope | Crustaceans (62%) | ^24^ |
|  |  | Demersal | Fish (18%) | ^25^ |
|  |  | 0 - 500 m | Worms | ^3^ |
| Lookdown dory  *Cyttus traversi* | 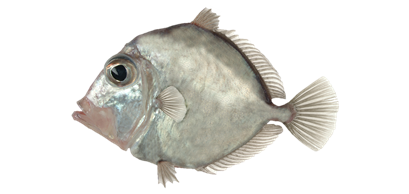700 | Slope | Crustaceans (82%) | ^1^ |
|  |  | Bathydemersal | Fish (20%) | ^26^ |
|  |  | 200 - 600 m | Cnidarians | ^27^ |
|  |  |  | Worms | ^28^ |
| Hoki *Macruronus novaezelandiae* | 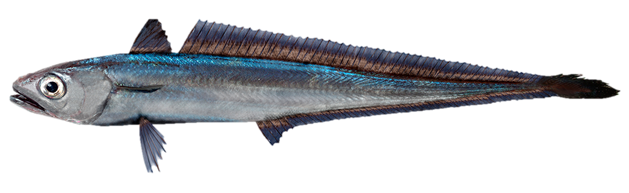1020 | Slope | Fish (60%) | ^5^ |
|  |  | Benthopelagic | Crustaceans (43%) | ^29^ |
|  |  | 200 - 600 m | Cephalopods (5%) | ^28^ |
|  |  |  | Sponges | ^30^ |
|  |  |  | Tunicates | ^31^ |
|  |  |  | Planktonic invertebrates |  |
| Hapuka *Polyprion oxygeneious* | 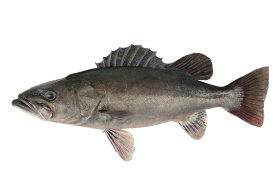1500 | Slope | Fish (68%) | ^32^ |
|  |  | Demersal | Cephalopods (25%) | ^33^ |
|  |  | 0 – 850 | Crustaceans (18%) | ^34^ |
| Ling *Genypterus blacodes* | 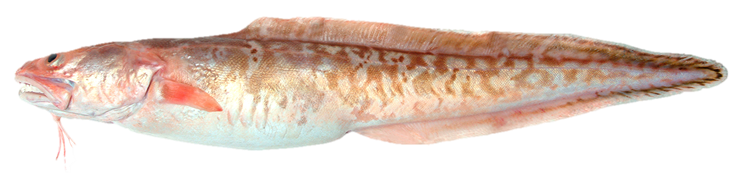2000 | Slope | Fish (65%) | ^35^ |
|  |  | Bathydemersal | Crustaceans (37%) | ^20^ |
|  |  | 0 - 1000 m | Cephalopods (3%) | ^36^ |
|  |  |  | Ophiuroids | ^3^ |
|  |  |  |  | ^37^ |
| Orange roughy *Hoplostethus atlanticus* | 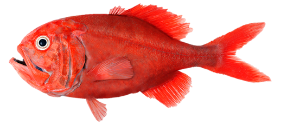500 | Mid shelf | Crustaceans (58%) | ^38^ |
|  |  | Bathypelagic | Fish (41%) | ^5^ |
|  |  | 700 - 1500 m | Cephalopods | ^39^ |
|  |  |  | Planktonic invertebrates |  |
|  |  |  | Worms |  |
|  | | | |  |
|  | | | |  |

**Table S2.**

Layman’s metrics calculated for the whole fish community and for specific assemblages (Table S1). Mean and 95% credible interval of metrics were calculated with Bayesian framework using the SIBER package in R ^40–42^ and applying corrected carbon and nitrogen isotope values of fish muscle tissue from historical and modern periods. Mid-slope assemblage is comprised by orange roughy only and therefore is not shown. Using the description used by Jackson *et al.*: Total area of convex hull (TA): indication of niche width; Mean distance from species to community centroid (CD): additional information on niche width and species spacing; Mean nearest neighborhood distance (MNND): measure of density and clustering of species within the community; Standard deviation of MNND (SDNND): measure of evenness of spatial density and packing; Standard Ellipse Area corrected for small sample sizes (SEA_c_): indication of niche width unbiased by different and small sample sizes. Bold values represent metrics that differed at the 95% credible interval between time periods.

|  |  | Mean | | 95% credible interval | |
| --- | --- | --- | --- | --- | --- |
| Assemblage | Metric | Historical | Modern | Historical | Modern |
| Whole community | δ¹⁵N range | 3.79 | 3.62 | 2.83 - 4.71 | 2.95 - 4.29 |
|  | δ¹³C range | 2.22 | 3.21 | 1.42 - 3.09 | 2.54 - 3.9 |
|  | TA* | 3.60 | 5.87 | 2.05 - 5.28 | 4.42 - 7.36 |
|  | CD | **0.91** | **1.25** | 0.73 - 1.1 | 1.1 - 1.41 |
|  | MNND | 0.40 | 0.46 | 0.29 - 0.51 | 0.22 - 0.58 |
|  | SDNND | 0.36 | 0.24 | 0.18 - 0.55 | 0.12 - 0.37 |
|  | TA** | 12.48 | 23.09 | - | - |
|  | SEAc** | **1.89** | **3.73** | 1.61 - 2.18 | 3.32 - 4.17 |
| Inner shelf | δ¹⁵N range | 1.71 | 1.63 | 0.9 - 2.53 | 0.91 - 2.41 |
|  | δ¹³C range | 1.20 | 1.46 | 0.37 - 2.07 | 0.59 - 2.42 |
|  | TA* | 0.77 | 1.00 | 0.1 - 1.48 | 0.36 - 1.72 |
|  | CD | 0.75 | 0.81 | 0.41 - 1.1 | 0.53 - 1.1 |
|  | MNND | 0.75 | 0.86 | 0.39 - 1.15 | 0.55 - 1.17 |
|  | SDNND | 0.35 | 0.36 | 0.01 - 0.71 | 0.01 - 0.73 |
|  | TA** | 10.21 | 11.00 | - | - |
|  | SEAc** | 2.55 | 2.27 | 1.79 - 3.34 | 1.78 - 2.79 |
| Outer shelf | δ¹⁵N range | 3.33 | 2.33 | 2.38 - 4.34 | 1.75 - 2.9 |
|  | δ¹³C range | 1.79 | 2.00 | 0.94 - 2.73 | 1.41 - 2.59 |
|  | TA* | 2.30 | 1.97 | 0.88 - 3.84 | 0.99 - 3 |
|  | CD | 1.06 | 0.94 | 0.78 - 1.37 | 0.73 - 1.15 |
|  | MNND | 0.83 | 0.75 | 0.57 - 1.1 | 0.49 - 1 |
|  | SDNND | 0.74 | 0.37 | 0.37 - 1.11 | 0.12 - 0.61 |
|  | TA** | 7.45 | 20.64 | - | - |
|  | SEAc** | **2.16** | **3.54** | 1.64 - 2.69 | 2.94 - 4.19 |
| Slope | δ¹⁵N range | 1.71 | 3.15 | 0.81 - 2.63 | 2.26 - 4.04 |
|  | δ¹³C range | 1.47 | 2.09 | 0.62 - 2.37 | 1.52 - 2.67 |
|  | TA* | **0.80** | **2.60** | 0.18 - 1.5 | 1.54 - 3.75 |
|  | CD | 0.76 | 1.19 | 0.41 - 1.13 | 0.93 - 1.48 |
|  | MNND | 0.56 | 1.00 | 0.25 - 0.9 | 0.7 - 1.3 |
|  | SDNND | 0.28 | 0.55 | 0.02 - 0.56 | 0.16 - 0.95 |
|  | TA** | 4.83 | 11.13 | - | - |
|  | SEAc** | **1.25** | **2.96** | 0.94 - 1.59 | 2.33 - 3.6 |

*Calculated between species centroids

**Calculated using all data points of each assemblage

**Table S3.**

Results from PERMANOVA analysis of interspecific value-predicted percentage phytoplankton supporting the food webs and trophic level between historical (before 1996) and modern (after 2000) periods. Numbers represent pseudo-F statistics calculated through at least 7281 unique permutations during PERMANOVA analysis, followed by its significance level.

|  | Percentage of phytoplankton | | |  | Trophic level | | |
| --- | --- | --- | --- | --- | --- | --- | --- |
| Species | Historical | Modern | Pseudo-F |  | Historical | Modern | Pseudo-F |
| Leatherjacket | 0.04 ± 0.01 | 0.0002 ± 0.0138 | 3.092 |  | -0.2 ± -0.04 | -0.0001 ± 0.0363 | 16.623*** |
| Blue cod | -0.06 ± -0.02 | -0.0001 ± 0.0107 | 4.059* |  | 0.05 ± 0.02 | 0.0005 ± 0.0369 | 0.32 |
| Gurnard | -0.05 ± -0.01 | 0.0014 ± 0.0078 | 4.497* |  | -0.3 ± -0.07 | -0.0003 ± 0.0256 | 58.833*** |
| Elephant fish | 0.11 ± 0.03 | 0.0002 ± 0.0379 | 2.97 |  | -0.26 ± -0.07 | -0.0001 ± 0.0111 | 2.262 |
| Common warehou | -0.01 ± -0.01 | 0.0005 ± 0.0118 | 0.282 |  | 0.03 ± 0.01 | -0.0003 ± 0.0482 | 0.242 |
| Barracouta | -0.03 ± -0.01 | -0.0002 ± 0.0113 | 1.378 |  | -0.16 ± -0.06 | 0.0002 ± 0.0308 | 1.821 |
| Tarakihi | -0.12 ± -0.03 | 0 ± 0.0159 | 14.133*** |  | -0.15 ± -0.03 | 0.0003 ± 0.0363 | 4.9* |
| Spiny dogfish | -0.07 ± -0.02 | 0 ± 0.0295 | 3.73 |  | -0.57 ± -0.16 | 0.0005 ± 0.0784 | 33.507*** |
| Giant stargazer | -0.01 ± 0 | -0.0014 ± 0.0078 | 0.153 |  | 0.23 ± 0.06 | -0.0003 ± 0.0599 | 4.34* |
| Red cod | 0.05 ± 0.01 | -0.0012 ± 0.0084 | 4.357* |  | -0.38 ± -0.1 | -0.0004 ± 0.026 | 46.188*** |
| Sea perch | -0.03 ± -0.01 | 0.0007 ± 0.008 | 3.784 |  | 0.34 ± 0.06 | -0.0004 ± 0.0418 | 37.343*** |
| Lookdown dory | -0.01 ± 0 | -0.0026 ± 0.0098 | 0.014 |  | 0.13 ± 0.03 | 0.0014 ± 0.0389 | 0.2802 |
| Hoki | -0.01 ± 0 | -0.0003 ± 0.0115 | 0.043 |  | -0.35 ± -0.11 | 0.0002 ± 0.0335 | 16.23** |
| Hapuka | 0.14 ± 0.05 | -0.0004 ± 0.026 | 8.657* |  | -0.48 ± -0.17 | 0.0031 ± 0.0389 | 18.652** |
| Ling | -0.12 ± -0.03 | 0.0047 ± 0.011 | 28.48*** |  | -0.2 ± -0.05 | 0 ± 0.0556 | 5.685* |
| Orange roughy | -0.06 ± -0.01 | 0.0001 ± 0.0087 | 1.275 |  | -0.54 ± -0.13 | -0.0003 ± 0.0376 | 40.555*** |

0.05(*), 0.005(**) and 0.0005(***) significance level

**Table S4.**

Results of generalized linear models (slope estimates ± SE) describing the effects of abundance of pelagic prey (MUN), temperature (SST), Southern Oscillation Index (SOI) and fisheries (MTI) on the variability of percentage phytoplankton supporting the food webs and trophic level for each fish assemblage. Significance of species random effects are also presented in the form of p values.

|  | Percentage phytoplankton | p value | Trophic level | p value |
| --- | --- | --- | --- | --- |
|  | (value-predicted ± SE) |  | (value-predicted ± SE) |  |
| **Inner shelf** | n = 181 | | n = 181 | |
| Intercept | 0.015 ± 0.021 | 0.509 | -0.152 ± 0.025 | **<0.0001** |
| MUN | 0.0001 ± 0.0007 | 0.909 | -0.008 ± 0.002 | **<0.0001** |
| SST | -0.008 ± 0.019 | 0.688 | -0.027 ± 0.052 | 0.607 |
| SOI | -0.048 ± 0.033 | 0.138 | 0.117 ± 0.086 | 0.176 |
| MTI | 0.076 ± 0.065 | 0.242 | -0.295 ± 0.169 | 0.089 |
| Random effect (Species) | 0.263 | | 0.695 | |
| Adjusted R² | 0.13 | | 0.196 | |
| **Outer shelf** | n = 220 | | n = 224 | |
| Intercept | -0.03 ± 0.014 | **0.05** | -0.126 ± 0.046 | **0.026** |
| MUN | -0.001 ± 0.001 | 0.055 | -0.005 ± 0.001 | **0.0003** |
| SST | 0.026 ± 0.021 | 0.213 | 0.042 ± 0.056 | 0.452 |
| SOI | -0.044 ± 0.024 | 0.071 | 0.055 ± 0.066 | 0.403 |
| MTI | -0.075 ± 0.071 | 0.29 | -0.381 ± 0.189 | 0.046 |
| Random effect (Species) | 0.359 | | 0.255 | |
| Adjusted R² | 0.057 | | 0.211 | |
| **Slope** | n = 172 | | n = 171 | |
| Intercept | -0.015 ± 0.026 | 0.585 | -0.027 ± 0.086 | 0.767 |
| MUN | 0.0002 ± 0.0005 | 0.6715 | -0.001 ± 0.001 | 0.6963 |
| SST | -0.003 ± 0.025 | 0.911 | 0.266 ± 0.07 | **0.0002** |
| SOI | 0.138 ± 0.018 | **<0.0001** | -0.366 ± 0.049 | **<0.0001** |
| MTI | 0.075 ± 0.092 | 0.416 | -1.213 ± 0.26 | **<0.0001** |
| Random effect (Species) | 0.279 | | 0.214 | |
| Adjusted R² | 0.358 | | 0.363 | |
| **Mid slope** | n = 33 | | n = 33 | |
| Intercept | -0.154 ± 0.073 | **0.044** | -0.759 ± 0.09 | **<0.0001** |
| MUN | 0.0001 ± 0.002 | 0.947 | 0.01 ± 0.002 | **0.001** |
| SST | 0.035 ± 0.063 | 0.581 | 0.691 ± 0.078 | **<0.0001** |
| SOI | -0.069 ± 0.09 | 0.452 | -0.292 ± 0.111 | **0.014** |
| MTI | 0.93 ± 0.623 | 0.147 | 2.467 ± 0.77 | **0.004** |
| Adjusted R² | 0.045 | | 0.76 | |

**Table S5.**

Predicted equations and their fit of percentage phytoplankton supporting the food webs and trophic level for fish species sampled in the modern period. Latitude (LAT) and total length were used as predictors, but only variables contributing significantly to the general linear model (p < 0.05) were kept. When percentage phytoplankton and trophic level could not be predicted by any of the variables an average of values was used as te predicted value in the present study (see Materials and Methods).

|  | Percentage phytoplankton | | | | |
| --- | --- | --- | --- | --- | --- |
| Species | Predictor equation/ average ± SD | Adjusted r² | ΔAICc | p value (TOTAL LENGTH) | p value (LAT) |
| Barracouta | 2.09667 + 0.03528 * LAT | 0.68 | 2.01 | - | 0.0001 |
| Blue cod | 0.573 ± 0.081 | - | - | - | - |
| Common warehou | 0.564 ± 0.039 | - | - | - | - |
| Ellephant fish | 0.335 ± 0.085 | - | - | - | - |
| Giant stargazer | 0.65207 + -0.00033 * TOTAL LENGTH | 0.42 | 2.63 | 0.003 | - |
| Gurnard | 0.68149 + -0.00079 * TOTAL LENGTH | 0.65 | 2.44 | <0.0001 | - |
| Hapuka | 0.486 ± 0.089 | - | - | - | - |
| Hoki | 0.708 ± 0.03 | - | - | - | - |
| Leatherjacket | 1.82423 + 0.03004 *LAT | 0.33 | - | - | 0.001 |
| Ling | 1.66216 + -0.00027*TOTAL LENGTH + 0.02082*LAT | 0.45 | 3.31 | 0.0002 | 0.021 |
| Lookdown dory | -15.89447 -0.00039*TOTA LENGTH + -0.38175 *LAT | 0.59 | 0.56 | 0.053 | 0.002 |
| Orange roughy | 0.696 ± 0.032 | - | - | - | - |
| Red cod | 2.03625 + -0.00052*TOTAL LENGTH + 0.02837*LAT | 0.61 | 10.32 | 0.0007 | 0.0005 |
| Sea perch | 1.80186 + -0.00094*TOTAL LENGTH + 0.02267*LAT | 0.37 | 9.9 | 0.0008 | 0.0001 |
| Spiny dogfish | 0.582 ± 0.121 | - | - | - | - |
| Tarakihi | 0.58 ± 0.119 | - | - | - | - |
|  |  |  |  |  |  |
|  | Trophic level | | | | |
| Species | Predictor equation/ average ± SD | Adjusted r² | ΔAICc | p value (TOTAL LENGTH) | p value (LAT) |
| Barracouta | 9.34184 + 0.14702 * LAT | 0.83 | 2.72 | - | <0.0001 |
| Blue cod | 3.079 ± 0.281 | - | - | - | - |
| Common warehou | 3.175 ± 0.159 | - | - | - | - |
| Ellephant fish | 2.974 ± 0.025 | - | - | - | - |
| Giant stargazer | 3.902 ± 0.247 | - | - | - | - |
| Gurnard | 3.431 ± 0.123 | - | - | - | - |
| Hapuka | -3.07412 + 0.00214 * TOTAL LENGH + -0.14006 *LAT | 0.53 | -0.27¹ | 0.011 | 0.023 |
| Hoki | 3.679 ± 0.089 | - | - | - | - |
| Leatherjacket | -0.33825 + -0.07838 *LAT | 0.33 | - | - | 0.001 |
| Ling | 14.32206 + 0.22977*LAT | 0.65 | 1.61 | - | <0.0001 |
| Lookdown dory | 65.25656 + 0.00158*TOTAL LENGTH + 1.44929 *LAT | 0.57 | 0.63 | 0.051 | 0.003 |
| Orange roughy | 3.476 ± 0.141 | - | - | - | - |
| Red cod | 3.228 ± 0.142 | - | - | - | - |
| Sea perch | 4.8621 + 0.00264*TOTAL LENGTH + 0.0674*LAT | 0.3 | 1.5 | 0.056 | 0.019 |
| Spiny dogfish | 2.882 ± 0.323 | - | - | - | - |
| Tarakihi | 3.116 ± 0.274 | - | - | - | - |

**Table S6.**

Linear relationship between head length (HL) and total length for species analyzed in the present study, with respective summary of fit.

| Common name | Total length regression equation | Sample size | | r^2^ | | | p |  |
| --- | --- | --- | --- | --- | --- | --- | --- | --- |
| Barracouta | 163.32 + 3.3*HL | | 32 | | 0.83 | <0.0001 | | |
| Blue cod | 26.36 + 3.5*HL | | 84 | | 0.98 | <0.0001 | | |
| Common warehou | -109.85 + 5.45*HL | | 12 | | 0.94 | <0.0001 | | |
| Elephant fish | 141.35 + 2.4*HL | | 9 | | 0.59 | 0.015 | | |
| Giant stargazer | 7.66 + 4.02*HL | | 6 | | 0.8 | 0.015 | | |
| Gurnard | 36.78 + 3.55*HL | | 27 | | 0.96 | <0.0001 | | |
| Hapuka | -40.26 + 2.8*HL | | 4 | | 0.99 | 0.0018 | | |
| Leatherjacket | 48.84 + 3.45*HL | | 29 | | 0.83 | <0.0001 | | |
| Ling | 150.81 + 3.8*HL | | 9 | | 0.86 | 0.0003 | | |
| Red cod | 127.16 + 3.47*HL | | 14 | | 0.52 | 0.0036 | | |
| Sea perch | 62.32 + 2.56*HL | | 56 | | 0.88 | <0.0001 | | |
| Spiny dogfish | 7.19 + 4.79*HL | | 20 | | 0.93 | <0.0001 | | |
| Tarakihi | -13.95 + 4.21*HL | | 38 | | 0.92 | <0.0001 | | |

**Table S7.**

Sample sizes used in Layman’s metrics analysis for all species collected in different regions during historical and modern time periods.

| Species | Region | Historical | Modern | Total |
| --- | --- | --- | --- | --- |
| Blue cod | All | 6 | 40 | 46 |
| *Parapercis colias* | Canterbury Bight | 3 | 23 | 26 |
|  | North of Banks Peninsula-Shallow |  | 4 | 4 |
|  | Top of the South Island | 3 | 13 | 16 |
| Elephant fish | All | 13 | 5 | 18 |
| *Callorhinchus milii* | Canterbury Bight | 13 | 5 | 18 |
| Gurnard | All | 16 | 21 | 37 |
| *Chelidonichthys kumu* | Canterbury Bight | 13 | 20 | 33 |
|  | North of Banks Peninsula-Shallow | 2 |  | 2 |
|  | Top of the South Island | 1 | 1 | 2 |
| Leatherjacket | All | 6 | 12 | 18 |
| *Meuschenia scaber* | Canterbury Bight | 6 | 12 | 18 |
| Barracouta | All | 8 | 15 | 23 |
| *Thyrsites atun* | Canterbury Bight | 5 | 11 | 16 |
|  | North of Banks Peninsula-Shallow | 1 |  | 1 |
|  | Top of the South Island | 2 | 4 | 6 |
| Common warehou | All | 6 | 11 | 17 |
| *Seriolella brama* | Canterbury Bight | 6 | 11 | 17 |
| Giant stargazer | All | 6 | 8 | 14 |
| *Kathetostoma giganteum* | Canterbury Bight | 6 | 8 | 14 |
| Red cod | All | 10 | 33 | 43 |
| *Pseudophycis bachus* | Canterbury Bight | 4 | 15 | 19 |
|  | North of Banks Peninsula-Deep | 3 | 8 | 11 |
|  | North of Banks Peninsula-Shallow | 2 | 9 | 11 |
|  | Top of the South Island | 1 | 1 | 2 |
| Spiny dogfish | All | 12 | 16 | 28 |
| *Squalus acanthias* | Canterbury Bight | 12 | 16 | 28 |
| Tarakihi | All | 21 | 45 | 66 |
| *Nemadactylus macropterus* | Canterbury Bight | 15 | 25 | 40 |
|  | North of Banks Peninsula-Shallow | 6 | 17 | 23 |
|  | Top of the South Island |  | 3 | 3 |
| Hapuka | All | 7 | 7 | 14 |
| *Polyprion oxygeneious* | Canterbury Bight | 3 | 7 | 10 |
|  | North of Banks Peninsula-Deep | 1 |  | 1 |
|  | North of Banks Peninsula-Shallow | 1 |  | 1 |
|  | Top of the South Island | 2 |  | 2 |
| Ling | All | 11 | 15 | 26 |
| *Genypterus blacodes* | Canterbury Bight | 9 | 5 | 14 |
|  | North of Banks Peninsula-Deep | 1 | 9 | 10 |
|  | Top of the South Island | 1 | 1 | 2 |
| Sea perch | All | 25 | 32 | 57 |
| *Helicolenus percoides* | Canterbury Bight | 18 | 21 | 39 |
|  | North of Banks Peninsula-Deep | 1 |  | 1 |
|  | North of Banks Peninsula-Shallow |  | 10 | 10 |
|  | Top of the South Island | 6 | 1 | 7 |
| Hoki | All | 9 | 7 | 16 |
| *Macruronus novaezelandiae* | Canterbury Bight | 2 |  | 2 |
|  | North of Banks Peninsula-Deep | 7 | 7 | 14 |
| Lookdown dory | All | 11 | 11 | 22 |
| *Cyttus traversi* | Canterbury Bight | 1 |  | 1 |
|  | North of Banks Peninsula-Deep | 10 | 11 | 21 |
| Orange roughy | All | 8 | 13 | 21 |
| *Hoplostethus atlanticus* | North of Banks Peninsula-Deep | 8 | 13 | 21 |
| Total |  | 175 | 291 | 466 |

**Table S8.**

Sample sizes used in trophic level and percentage phytoplankton supporting the fish community analysis for all species collected in different regions during historical and modern time periods.

| Species | Region | Historical | Modern | Total |
| --- | --- | --- | --- | --- |
| Blue cod | All | 11 | 58 | 69 |
| *Parapercis colias* | Canterbury Bight | 5 | 30 | 35 |
|  | North of Banks Peninsula-Shallow |  | 9 | 9 |
|  | Top of the South Island | 6 | 19 | 25 |
| Elephant fish | All | 15 | 5 | 20 |
| *Callorhinchus milii* | Canterbury Bight | 13 | 5 | 18 |
|  | North of Banks Peninsula-Shallow | 1 |  | 1 |
|  | Top of the South Island | 1 |  | 1 |
| Gurnard | All | 18 | 23 | 41 |
| *Chelidonichthys kumu* | Canterbury Bight | 15 | 22 | 37 |
|  | North of Banks Peninsula-Shallow | 2 |  | 2 |
|  | Top of the South Island | 1 | 1 | 2 |
| Leatherjacket | All | 23 | 26 | 49 |
| *Meuschenia scaber* | Canterbury Bight | 20 | 20 | 40 |
|  | North of Banks Peninsula-Shallow | 3 |  | 3 |
|  | Top of the South Island |  | 6 | 6 |
| Barracouta | All | 8 | 15 | 23 |
| *Thyrsites atun* | Canterbury Bight | 5 | 11 | 16 |
|  | North of Banks Peninsula-Shallow | 1 |  | 1 |
|  | Top of the South Island | 2 | 4 | 6 |
| Common warehou | All | 8 | 11 | 19 |
| *Seriolella brama* | Canterbury Bight | 6 | 11 | 17 |
|  | Top of the South Island | 2 |  | 2 |
| Giant stargazer | All | 16 | 17 | 33 |
| *Kathetostoma giganteum* | Canterbury Bight | 7 | 17 | 24 |
|  | North of Banks Peninsula-Deep | 2 |  | 2 |
|  | North of Banks Peninsula-Shallow | 2 |  | 2 |
|  | Top of the South Island | 5 |  | 5 |
| Red cod | All | 14 | 30 | 44 |
| *Pseudophycis bachus* | Canterbury Bight | 4 | 13 | 17 |
|  | North of Banks Peninsula-Deep | 7 | 8 | 15 |
|  | North of Banks Peninsula-Shallow | 2 | 9 | 11 |
|  | Top of the South Island | 1 |  | 1 |
| Spiny dogfish | All | 12 | 17 | 29 |
| *Squalus acanthias* | Canterbury Bight | 12 | 17 | 29 |
| Tarakihi | All | 21 | 57 | 78 |
| *Nemadactylus macropterus* | Canterbury Bight | 15 | 28 | 43 |
|  | North of Banks Peninsula-Shallow | 6 | 26 | 32 |
|  | Top of the South Island |  | 3 | 3 |
| Hapuka | All | 8 | 12 | 20 |
| *Polyprion oxygeneious* | Canterbury Bight | 4 | 7 | 11 |
|  | North of Banks Peninsula-Deep | 1 |  | 1 |
|  | North of Banks Peninsula-Shallow | 1 |  | 1 |
|  | Top of the South Island | 2 | 5 | 7 |
| Ling | All | 20 | 27 | 47 |
| *Genypterus blacodes* | Canterbury Bight | 17 | 8 | 25 |
|  | North of Banks Peninsula-Deep | 1 | 11 | 12 |
|  | Top of the South Island | 2 | 8 | 10 |
| Sea perch | All | 32 | 38 | 70 |
| *Helicolenus percoides* | Canterbury Bight | 24 | 24 | 48 |
|  | North of Banks Peninsula-Deep | 1 |  | 1 |
|  | North of Banks Peninsula-Shallow | 1 | 10 | 11 |
|  | Top of the South Island | 6 | 4 | 10 |
| Hoki | All | 11 | 7 | 18 |
| *Macruronus novaezelandiae* | Canterbury Bight | 4 |  | 4 |
|  | North of Banks Peninsula-Deep | 7 | 7 | 14 |
| Lookdown dory | All | 18 | 12 | 30 |
| *Cyttus traversi* | Canterbury Bight | 6 |  | 6 |
|  | North of Banks Peninsula-Deep | 12 | 12 | 24 |
| Orange roughy | All | 18 | 14 | 32 |
| *Hoplostethus atlanticus* | Canterbury Bight | 6 |  | 6 |
|  | North of Banks Peninsula-Deep | 12 | 14 | 26 |
| Total |  | 253 | 369 | 622 |

**References**

1. Anderson, O. F. *et al.* *Atlas of New Zealand Fish and Squid Distributions From Research Bottom Trawls. NIWA Technical Report 42*. (1998).

2. Russell, B. C. The food and feeding habits of rocky reef fish of north‐eastern New Zealand. *New Zealand Journal of Marine and Freshwater Research* **17**, 121–145 (1983).

3. Graham, D. H. Food of fishes of Otago Harbour and Adjacent Sea. *Royal Society of New Zealand* 421–436 (1939).

4. Carbines, G. & Mckenzie, J. *Movement patterns and stock mixing of blue cod in Southland (BC05). Ministry of Fisheries Research Project BCO9702*. (2001).

5. Fisheries New Zealand. *Fisheries Assessment Plenary, May 2019: stock assessments and stock status*. (2019).

6. Godfriaux, B. L. Food of predatory demersal fish in Hauraki Gulf. *New Zealand Journal of Marine and Freshwater Research* **4**, 325–336 (1970).

7. Stevens, D. W., Hurst, R. J. & Bagley, N. W. *Feeding habits of New Zealand fishes : a literature review and summary of research trawl database records 1960 to 2000. New Zealand Aquatic Environment and Biodiversity Report No. 85*. (2011).

8. Coleman, N. & Mobley, M. Diets of commercially exploited fish from Bass Strait and adjacent Victorian Waters, south-eastern Australia. *Marine and Freshwater Research* **35**, 549–560 (1984).

9. Bagley, N. W., Ballara, S. L., Horn, P. L. & Hurst, R. J. *A summary of commercial landings and a validated ageing method for blue warehou,* Seriolella brama *(Centrolophidae), in New Zealand waters, and a stock assessment of the Southern (WAR 3) Fishstock. New Zealand Fisheries Assessment Research Document* . (1998).

10. O’Driscoll, R. L. Feeding and schooling behaviour of barracouta (Thyrsites atun) off Otago, New Zealand. *Marine and Freshwater Research* **49**, 21–24 (1998).

11. Hurst, R. J., Ballara, S. L. & Macgibbon, D. *Fishery characterisation and standardised CPUE analyses for 1989 – 90 to 2007 – 08 Private Bag 14901 Wellington New Zealand New Zealand Fisheries Assessment Report 2012 / 12*. (Ministru of Agriculture and Forestry, 2012).

12. Godfriaux, B. L. Food of Tarakihi in Western bay of plenty and Tasman bay, New Zealand. *New Zealand Journal of Marine and Freshwater Research* **8**, 111–153 (1974).

13. McKenzie, J. R. *et al.* *Fishery characterisation and age composition of tarakihi in TAR 1, 2 and 3 for 2013/14 and 2014/15. New Zealand Fisheries Assessment Report 2017/36*. (2017).

14. Annala, J. H. *New Zealand Fisheries Assessment Research Document 88/28*. (1988).

15. Cortés, E. Standardized diet compositions and trophic levels of sharks. *ICES Journal of Marine Science* **56**, 707–717 (1999).

16. Ellis, J. R., Pawson, M. G. & Shackley, S. E. The comparative feeding ecology of six species of shark and four species of ray (Elasmobranchii) in the North-East Atlantic. *Journal of the Marine Biological Association of the United Kingdom* **76**, 89–106 (1996).

17. Ebert, D. A., Compagno, L. J. V. & Cowley, P. D. A preliminary investigation of the feeding ecology of squaloid sharks off the west coast of southern Africa. *South African Journal of Marine Science* **12**, 601–609 (1992).

18. Fuita, T. *et al.* Diets of the demersal fishes on the shelf off Iwate, northern Japan. *Marine Biology* **123**, 219–233 (1995).

19. Demirhan, S. A. & Seyhan, K. Life history of spiny dogfish, *Squalus acanthias* (L. 1758), in the southern Black Sea. *Fisheries Research* **85**, 210–216 (2007).

20. Stevenson, M. L. *Trawl survey of the west coast of the South Island and Tasman and Golden Bays, March-April 2003 (KAHO304) - New Zealand Fisheries Assessment Report 200414*. (2004).

21. Sutton, C. P. *Estimation of age, growth, and mortality of giant stargazer (*Kathetostoma giganteurn*) from Southland trawl surveys between 1993 and 1996. New Zealand Fisheries Assessment Report 2004/38*. (2004).

22. Cohen, D. M., Inada, T., Iwamoto, T. & Scialabba, N. FAO species catalogue. Gadiform fishes of the world (Order Gadiformes). *An annotated and illustrated catalogue of cods, hakes, grenadiers and other gadiform fishes known to date.* 442 (1990).

23. Edgar, G. J. & Shaw, C. The production and trophic ecology of shallow-water fish assemblages in southern Australia II. Diets of fishes and trophic relationships between fishes and benthos at Western Port, Victoria. *Journal of Experimental Marine Biology and Ecology* **194**, 83–106 (1995).

24. Paulin, C. D. Redescription of *Helicolenus percoides* (Richardson) and *H. barathri* (Hector) from New Zealand (Pisces, Scorpaenidae). *Journal of the Royal Society of New Zealand* **19**, 319–325 (1989).

25. Smith, P. *Molecular identification of sea perch species. Final Report to the Ministry of Fisheries for Project MOF706 - Unpublished report*. (1998).

26. Tracey, D. M., Horn, P. L., Andrews, A. H., Marriott, P. M. & Dunn, M. R. *Age and growth, and an investigation of age validation of lookdown dory (*Cyttus traversi*). Final Research Report for Ministry of Fisheries Research Project LDO2004-01*. (2007).

27. Forman, J. & Dunn, M. The influence of ontogeny and environment on the diet of lookdown dory, *Cyttus traversi*. *New Zealand Journal of Marine and Freshwater Research* **44**, 329–342 (2010).

28. Blaber, S. J. M. & Bulman, C. M. Diets of fishes of the upper continental slope of eastern Tasmania: content, calorific values, dietary overlap and trophic relationships. *Marine Biology* **95**, 345–356 (1987).

29. Horn, P. L. & Sullivan, K. J. Validated aging methodology using otoliths, and growth parameters for hoki (*Macruronus novaezelandiae*) in New Zealand waters. *New Zealand Journal of Marine and Freshwater Research* **30**, 161–174 (1996).

30. Bulman, C. & Blaber, S. Feeding ecology of *Macruronus novaezelandiae* (Hector) (Teleostei : Merlucciidae) in south-eastern Australia. *Marine and Freshwater Research* **37**, 621 (1986).

31. Clark, M. R. Feeding relationships of seven fish species from the Campbell Plateau, New Zealand. *New Zealand Journal of Marine and Freshwater Research* **19**, 365–374 (1985).

32. Paxton, J. R., Hoese, D. F., Allen, G. R. & Hanley, J. E. *Zoological Catalogue of Australia: Pisces: Petromyzontidae to Carangidae (Zoological Catalogue of Australia)*. (CSIRO Publishing, 1989).

33. Saldanha, L. *Fauna submarina atlântica: Portugal continental, Açores, Madeira*. (Mem Martins: Publicac̜ões Europa-América., 1997).

34. Rojas, P., Flores, H. & Sepúlveda, J. Alimentación del Bacalao de Juan Fernández *Polyprion oxygeneios* (Bloch y Scneider , 1801) (Pisces : Percichthyidae). *Investigaciones Marinas en el Archipiélago de Juan Fernández* 305–309 (1985).

35. Nielsen, J. G., Cohen, D. M., Markle, D. F. & Robins, C. R. *Ophidiiform fishes of the world (Order Ophidiiformes). An annotated and illustrated catalogue of pearlfishes, cusk-eels, brotulas and other ophidiiform fishes known to date.* *FAO Fish. Synop.* (The Food and Agriculture Organization of the United Nations, 1999).

36. Dunn, M. R., Connell, A. M., Forman, J., Stevens, D. W. & Horn, P. L. Diet of Two Large Sympatric Teleosts, the Ling (*Genypterus blacodes*) and Hake (*Merluccius australis*). *PLoS ONE* **5**, 1–11 (2010).

37. Kailola, P. J. *et al.* *Australian Fisheries Resources*. (Bureau of Resource Sciences, Department of Primary Industries and Energy, Australia, 1993).

38. Gordon, J. D. M. & Duncan, J. A. R. Aspects of the biology of *Hoplostethus atlanticus* and *H. mediterraneus* (Pisces: Berycomorphi) from the slopes of the rockall Trough and the Porcupine Sea Bight (north-eastern Atlantic). *Journal of the Marine Biological Association of the United Kingdom* **67**, 119–133 (1987).

39. Bulman, C. & Koslow, J. Diet and food consumption of a deep-sea fish orange roughy *Hoplostethus atlanticus* (Pisces Trachichthyidae), off southeastern Australia. *Marine Ecology Progress Series* **82**, 115–129 (1992).

40. R Core Team. R: A language and environment for statistical computing. (2020).

41. Layman, C. A., Arrington, D. A., Montaña, C. G. & Post, D. M. Can stable isotope ratios provide for community-wide measures of trophic structure? *Ecology* **88**, 42–48 (2007).

42. Jackson, A. L., Inger, R., Parnell, A. C. & Bearhop, S. Comparing isotopic niche widths among and within communities: SIBER - Stable Isotope Bayesian Ellipses in R. *Journal of Animal Ecology* **80**, 595–602 (2011).
